# *Helicobacter pylori* CagA: From Pathogenic Mechanisms to Its Use as an Anti-Cancer Vaccine

**DOI:** 10.3389/fimmu.2013.00328

**Published:** 2013-10-15

**Authors:** Markus Stein, Paolo Ruggiero, Rino Rappuoli, Fabio Bagnoli

**Affiliations:** ^1^Albany College of Pharmacy and Health Sciences, Albany, NY, USA; ^2^Research Center, Novartis Vaccines, Siena, Italy

**Keywords:** *Helicobacter pylori*, CagA, epithelial to mesenchymal transition, vaccine, junctions, cancer, type IV secretion system

## Abstract

*Helicobacter pylori* colonizes the gastric mucosa of more than 50% of the human population, causing chronic inflammation, which however is largely asymptomatic. Nevertheless, *H. pylori*-infected subjects can develop chronic gastritis, peptic ulcer, gastric mucosa-associated lymphoid tissue lymphoma, and gastric cancer. Chronic exposure to the pathogen and its ability to induce epithelial to mesenchymal transition (EMT) through the injection of cytotoxin-associated gene A into gastric epithelial cells may be key triggers of carcinogenesis. By deregulating cell–cell and cell–matrix interactions as well as DNA methylation, histone modifications, expression of micro RNAs, and resistance to apoptosis, EMT can actively contribute to early stages of the cancer formation. Host response to the infection significantly contributes to disease development and the concomitance of particular genotypes of both pathogen and host may turn into the most severe outcomes. T regulatory cells (Treg) have been recently demonstrated to play an important role in *H. pylori*-related disease development and at the same time the Treg-induced tolerance has been proposed as a possible mechanism that leads to less severe disease. Efficacy of antibiotic therapies of *H. pylori* infection has significantly dropped. Unfortunately, no vaccine against *H. pylori* is currently licensed, and protective immunity mechanisms against *H. pylori* are only partially understood. In spite of promising results obtained in animal models of infection with a number of vaccine candidates, few clinical trials have been conducted so far and with no satisfactory outcomes. However, prophylactic vaccination may be the only means to efficiently prevent *H. pylori*-associated cancers.

## Introduction

*Helicobacter pylori*, since its culture from a gastric biopsy in 1982 (1), has become one of the most studied bacteria with a number of publications comparable to those on *Staphylococcus* and *Mycobacterium* genus, which are second only to *Escherichia coli*, the most cited bacterial species. Research on *H. pylori* had been truly global due to the interest of investigators from many disciplines, including microbiologists, gastroenterologists, cancer biologists, and those in pharmaceutical industry.

*Helicobacter pylori* is a spiral-shaped, flagellated, micro-aerophilic Gram-negative bacillus that colonizes the gastric mucosa of more than 50% of the human population, with the highest prevalence in developing countries (2, 3). The infection is transmitted within the family in childhood (4, 5), likely by fecal-oral transmission (6, 7). A recent meta-analysis related the presence of *H. pylori* in the oral cavity to gastric colonization and possible reinfection (6, 7). *H. pylori* presence in tonsils is controversial (8–10); if confirmed, it could help further understanding of *H. pylori* transmission and reinfection. An updated review on the *H. pylori* epidemiology is presented in (11).

*Helicobacter pylori* is the etiological agent of severe gastric diseases. In particular, a subset of the colonized individuals may develop corpus gastritis, gastric atrophy, gastric ulcer, and increased risk of gastric cancer, whereas another subset may develop antral-predominant gastritis, associated with gastric hyperchlorhydria and increased risk of duodenal ulcer (12–15).

In 1994 the International Agency for Research on Cancer (IARC) identified *H. pylori* as a group 1 carcinogen (75% attributable risk) on the basis of epidemiological data (16). Research concerning the association with gastric cancer has achieved enormous progress over time, and molecular pathogenesis studies are providing strong evidences for an active role of the bacterium.

In the majority of *H. pylori*-infected population, however, infection results in asymptomatic chronic active gastritis. Symptomatic diseases occur in approximately only 10% of infected individuals. The explanation of such a phenomenon may reside on host factors, such as genetic predisposition to higher colonization and to inflammatory response. Furthermore, epidemiological studies suggest that *H. pylori* strain-specific virulence factors play a major role in the pathogenesis. One of the best characterized toxins of *H. pylori* is cytotoxin-associated gene A (CagA), the product of *cag*A which is associated with enhanced induction of gastritis, peptic ulcer, and higher risk of gastric cancer (17–21).

The present manuscript focuses on the interaction between *H. pylori*, and in particular CagA, with host cell, molecular mechanisms behind its association to gastric cancer, and on the potential role of vaccines in preventing such a deadly disease.

### Introducing *H. pylori*-associated malignancies

While various viruses have been successfully linked to human cancer, the oncogenic potential of bacteria remains less defined (22). *H. pylori* is the only bacterium to date that has been clearly associated with development of cancer (16). Several studies in animal models provided the formal evidence that *H. pylori* infection is able to promote cancer development (23–25).

According to the World Health Organization and the National Cancer Institute, gastric cancer is only second to lung carcinoma in terms of cancer-related mortality with 738,000 deaths annually and the fourth most common form of cancer (7.8%) overall (26).

A recent update of the IARC *H. pylori* monograph included a detailed overview of several studies on the association between *H. pylori* infection and various types of cancer (23). It must be noted that in some cases there is lack of agreement among the conclusions of different studies. This contradictory data makes it, for the moment, impossible to reach definitive statements about the association of *H. pylori* infection with certain cancer diseases. On the other hand, it can be stated that these contradictory results might be often due to the different geographic areas in which the studies were conducted, thus referring to subjects having different genetic background and also different lifestyle, which include diet and environmental conditions that can influence the outcome of the infection and the disease. A further element that could have influenced the outcome of those studies is constituted by the different methods used to assess *H. pylori* positivity of the subjects included in the studies. The IARC data on *H. pylori* and cancers are summarized in the following part of this paragraph.

The relationship of *H. pylori* infection with non-cardia gastric carcinoma (i.e., in the stomach region distal to the esophageal sphincter) is considered well established, with odds ratios (ORs) ranging from 1.07 to 21.0. In particular, association between CagA-positive strains and non-cardia gastric carcinoma was found. Several studies found that, among the *H. pylori*-infected subjects, the smoking habit, as well as diets including salted, smoked foods, and processed meats, significantly increase the risk of non-cardia gastric carcinoma; conversely, diets rich in fresh vegetables reduce the risk of non-cardia gastric carcinoma in *H. pylori*-infected subjects. Differently from non-cardia gastric carcinoma, association of *H. pylori* infection with cardia gastric carcinoma appears controversial, even when considering the CagA status.

Association of *H. pylori* infection with gastric mucosa-associated lymphoid tissue (MALT) lymphoma is considered proven by the fact that the eradication treatment of *H. pylori* infection consistently results in remission of MALT lymphoma (27).

Based on the epidemiological studies, there is no association between *H. pylori* infection and increased risk of esophageal adenocarcinoma; moreover, some of these studies, in contrast with others, indicate the reduction of risk of esophageal adenocarcinoma for *H. pylori*-infected subjects.

Liver cancers have been also evaluated for their possible association with *H. pylori*. Although such association was proposed with hepatocellular carcinoma and cholangiocarcinoma, the size of the available studies are considered too small to reach a definitive conclusion. Moreover, at least in the case of hepatocellular carcinoma, the conclusions of different studies are not in agreement.

Some studies reported association between *H. pylori* infection and colorectal cancer, cancer of the pancreas, and cancer of the lung (but in this case the studies were not adjusted for smoking habit). However, other studies did not find such significant relationships. Among the cancers of the head and neck, significant association with *H. pylori* infection was reported for squamous cells laryngeal cancer and squamous cell cancer of upper aerodigestive tract (excluding the esophagus), while moderate association was found for squamous cell carcinoma of the laryngopharynx. No association of *H. pylori* infection was found with childhood leukemia.

## Part 1 – Cellular and Molecular Mechanisms Associated with Cancer Induction by *H. pylori*

*Helicobacter pylori* has been linked to a myriad of cancer-related pathways *in vitro* and *in vivo* that provide a rationale for its ability to transform cells and cause malignancies (28–30).

**Figure 1:**
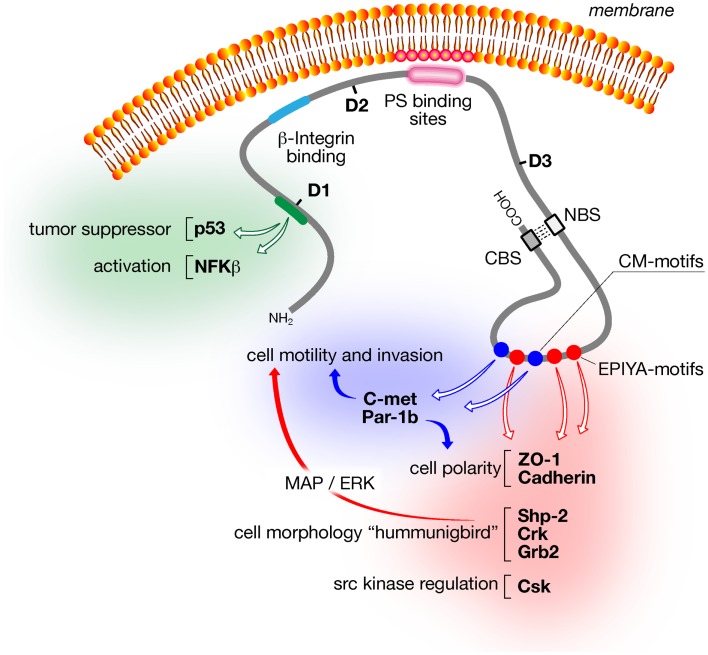
**Schematic of major CagA structural domains and functions**. The amino-terminal domain (D1; aa24–aa221) of CagA activates inflammatory responses via NFκB and prevents apoptosis via the tumor suppressor p53. The central domain (D2; aa303–aa644) contains the β1-integrin binding domain, which is required for CagA translocation into the host cell, and a segment of basic aa that tethers CagA to phosphatidylserine in the inner leaflet of the cytoplasmic membrane. The N-terminal binding sequence (NBS) located within the D3 domain (aa645–aa824) binds to the C-terminal binding sequence (CBS) located within the intrinsically unstructured C-terminus to form a loop-like structure that exposes the CM dimerization motifs (blue circles) and the EPIYA motifs (red circles). Both motifs trigger many of the CagA-dependent signaling events including disruption of cell polarity, morphological changes, cell motility, and invasion.

The presence of a pathogenicity island (*cag*) renders *H. pylori* more virulent (21, 31, 32). Encoded in *cag* is a type IV secretion system (T4SS), which is made up of circa 20 Cag proteins, and its substrate, the CagA (21). Although the role of other *H. pylori* proteins, such as the vacuolating toxin A (VacA) in cancer has been discussed, the CagA protein appears to be the major disease specific bacterial factor in cancer development (Figure 1) (33).

### CagA – a bacterial oncoprotein

Cytotoxin-associated gene A was identified as a cancer-associated factor long before its function was scrutinized, since isolates from cancer patients frequently expressed CagA, while strains from asymptomatic individuals or patients suffering from mild gastritis did not (34). Indeed, transgenic expression of CagA in mice was recently shown to cause multiple malignancies including gastric epithelial hyperplasia and, in some cases, gastric polyps and adenocarcinomas of the stomach and small intestine, or myeloid leukemias and B cell lymphomas, establishing the role of CagA as a bacterial oncoprotein (35). This study also demonstrated the relevance of CagA phosphorylation to the development of *H. pylori*-associated neoplasms, since mice expressing non-phosphorylable CagA did not present pathological abnormalities (23).

Up to this date, CagA is the only identified protein substrate of the T4SS and is delivered into the host cell during bacterial attachment to the gastric epithelial cell layer (36–39). CagA translocation requires binding of CagL, which is exposed on the surface of the secretion pilus structure, to the α5β1-integrin located on the basolateral surface of the cells (40–42). In addition, an amino-terminal region of CagA was also shown to bind to α5β1-integrin and is involved in CagA internalization into the host cell (43). Therefore, only bacteria that have reached the paracellular space and the lamina propria have access to the receptor and indeed *H. pylori* has been shown to colonize these intercellular niches (44). While it was originally thought that CagA uses the T4SS as a conduit to pass from the bacterial cytosol directly into the cytoplasm of the host cell, recent findings by Murata-Kamiya et al. demonstrated T4SS-dependent localization of CagA to the bacterial surface (45). Here CagA interacts with phosphatidylserine of the host, which is externalized from the inner to the outer leaflet of the plasma membrane as response to bacterial contact. CagA binding to phosphatidylserine requires the K-Xn-R-X-R motif within the central region of CagA. This interaction is followed by an as yet uncharacterized eukaryotic uptake mechanism. The K-Xn-R-X-R motif is conserved in various PH domains that are known to bind to acidic phospholipids. Inside the host cell, CagA is tethered to the inner surface of the plasma membrane, once again by the interaction between the central K-Xn-R-X-R motif of CagA with phosphatidylserine (45). Recent structural data indicate that CagA contains three distinct domains: a structural N-terminus followed by the phosphatidylserine binding domain, and a third domain that interacts intramolecularly with an intrinsically disordered C-terminal region (46).

Following translocation, Src-family and Abl kinases then phosphorylate CagA on tyrosine residues within a specific motif, EPIYA, which is found as part of a repetitive sequence within the carboxyterminal region of the protein (39, 47, 48). Following these events, CagA interacts with various host signaling factors and triggers cancer-related pathways that can be broken up into two categories, those that depend on tyrosine phosphorylation of the EPIYA motif [SHP-2, C-terminal Src kinase (CSK), Crk, E-cadherin] and those that depend on CagA, but are phosphorylation-independent (Grb2, c-Met, Par-1b/MARK2, ZO-1) (see below).

### Cell motility and proliferation

The first molecule that was shown to interact with phosphorylated CagA was the protooncogen SHP-2, a tyrosine phosphatase that links growth factor signaling with activation of Erk (49). Erk is part of the mitogen-activated protein kinase (MAPK) signaling pathway, which has been reported to play a role in carcinogenesis by inducing mitogenic responses (50). Indeed, CagA binding to the Src-homology domain 2 (SH2) of SHP-2 caused aberrant activation of SHP-2 and consequently of the ERK-MAPK pathway. Activation of this pathway by CagA may therefore act in enhanced cell-cycle progression and increase cell proliferation (51). In addition, SHP-2 activation also resulted in morphological changes, which have been described in AGS (human gastric adenocarcinoma) tissue culture cells as the “hummingbird phenotype” (37). This phenotype is characterized by dramatic cellular elongations and cytoskeletal rearrangements resembling those that occur during hepatocyte growth factor receptor (HGFR) activation (52). *In vitro*, these morphological changes also coincide with increased cell motility and a tendency of cells to detach from culture dishes. The search for SHP-2 phosphatase substrates that may explain CagA morphogenic activity led to the identification of focal adhesion kinase (FAK), a known regulator of the turnover of focal adhesions and cellular motility (53). FAK dephosphosphorylation via activated SHP-2 reduced FAK activity leading to a reduction of focal adhesion sites and contributing to cell detachment and increased cell motility. In agreement with these findings, reduced phosphorylation of FAK substrates like paxillin, which are involved in cell adhesion processes, was reported (53).

Interactions of CagA with additional signaling proteins that may contribute to cell morphological changes, cell substrate adhesion, and increased cell motility and proliferation have been described. These factors include Csk (54) the adapter proteins Grb2 (55) and Crk (56), and HGFR (c-Met) (57). Inhibition of Csk by phosphorylated CagA was shown to contribute to reduced cell–matrix adhesion by reducing the phosphorylation state of vinculin (54, 58). Vinculin was shown to be a major factor contributing to cell spreading and reduced wound healing. Thus both signals, CagA phosphorylation-dependent inhibition of FAK and Csk, seem to reduce cell adherence to the extracellular matrix (ECM) by causing dephosphorylation of cytoskeletal and FAK-associated proteins. Grb2 binding was suggested to act as a transducer of growth factor-like stimuli further contributing to the hummingbird phenotype and also to promote cell proliferation (55). These effects depended on activation of the Ras/MEK/ERK by CagA, but did not require CagA phosphorylation. Interaction of phosphorylated CagA with Crk was also shown to contribute to cell proliferation via Erk activation, albeit by a different signaling cascade than Grb2 (56). Additionally, Crk2 caused cytoskeletal changes by promoting Rac1 activity through the Crk/Dock180/ELMO pathway. Finally, the c-Met oncogene was activated by CagA independently of the CagA tyrosine-phosphorylation status. Activation of c-Met was suggested to deregulate growth factor receptor signaling and to play a role in mobility and invasiveness of gastric cells (57).

### Epithelial barrier function

Early studies by light and electron microscopy of stomach biopsy specimens demonstrated that *H. pylori* accumulates in two locations: within the gastric mucus and associated with intercellular junctions of gastric epithelial cells (59, 60). Human gastric mucosa of patients with gastric ulcers can show discontinuity and decrease in numbers of tight junctional strands, and *H. pylori* has been found around intercellular junctions with abnormalities of the tight-junction complexes (61). Despite these observations, the significance of this localization was unclear. More recently, *H. pylori* was demonstrated to interact with tight-junction components explaining the preferential localization of the bacterium at the cell–cell contacts observed in human mucosa (62). *H. pylori* uses CagA to attach near the intercellular junctions and disrupt the organization and function of the apical junctional complex (AJC) of cultured epithelial cells (62). AJC of epithelial cells form the barrier between the lumen and the interstitial space, and they also regulate several basic epithelial functions, such as the establishment of apical and basal polarity, cell proliferation, cell–cell adhesion, and cell movement. Independently of tyrosine phosphorylation, the N-terminus of CagA targets the protein to the epithelial junctions (63). Here, it complexes with several junction proteins and can perturb the assembly and function of both the tight and the adherens junctions (62–64). Phenotypically, this leads to the deregulation of epithelial cell–cell adhesion and loss of epithelial polarity. Recently, these effects of CagA on host cell polarity have been linked to the ability of *H. pylori* to colonize the surface of the host epithelial cell (65).

Evidence for how CagA causes disruption of cellular polarity on the molecular level came from observations that non-phosphorylated CagA interacts with the serine-threonine kinase Par-1b (MARK2) (64, 66). In polarized epithelial cells Par-1b is an essential component of the Par-aPKC system, which plays an important role in establishing cellular polarity by phosphorylation of various cellular targets including microtubule-associated proteins (MAPs) (67–69). CagA induced disruption of apical-basolateral polarity by inhibiting Par-1b kinase activity at the lateral cortex of MDCK polarized cell (66). In agreement, overexpression of Par-1b antagonized CagA-induced polarization defects. The FPLKRHDKVDDLSK peptide, also described as the CM (CagA multimerization) motif, which is located downstream of the EPIYA motifs in the C-terminal part of CagA, was sufficient to bind to the kinase substrate binding site of Par-1b and cause Par-1b inhibition by acting as a structural analog of kinase substrates (70). Association of CagA with Par-1b in MDCK cells not only caused disruption of tight junctions, but also prevented lumen formation and tubulogenesis, which are important hallmarks of epithelial differentiation (64). Furthermore inhibition of Par-1b kinase activity contributed to an increased hummingbird phenotype by acting on the actin cytoskeletal system (71). The authors demonstrated that inhibition of Par-1b prevented Par-1b-mediated phosphorylation and thus inactivation of the RhoA specific guanosine exchange factor GEF-H1, which is known to cause cortical actin and stress fiber formation and cell motility (72).

Another important function exerted by the CM motif of CagA is the association with E-cadherin (73–75). E-cadherin is a calcium-dependent cell–cell adhesion glycoprotein, which is crucial for the establishment of epithelial architecture as well as for maintenance of cell polarity and differentiation. Loss of expression of E-cadherin and disruption of the β-catenin/E-cadherin complex is considered an important factor in tumor development and loss or aberrant localization of E-cadherin is observed at sites of epithelial to mesenchymal transition (EMT) during tumor progression (76–78). While cell-to-cell interaction is mediated by homophilic E-cadherin interactions through the amino-terminal extracellular domain, the cytoplasmic carboxyterminus is linked to the actin cytoskeleton via α, β, and γ-catenins. In addition to stabilizing cell-to-cell adhesion, the cadherin-catenin complex is also a key regulator of the Wnt signaling pathway. In the absence of Wnt, β-catenin is modified by serine-phosphorylation, which causes its ubiquitination and subsequent degradation by the proteasomal complex. In the presence of Wnt, however, β-catenin phosphorylation is inhibited and β-catenin accumulates in the cytoplasm and gains access to the nucleus, where it induces transcription of various cancer-related genes, including NFAT (79, 80). CagA was shown to directly bind to E-cadherin and this interaction prevented association of E-cadherin with β-catenin causing destabilization of the adherence junction complex and redistribution of β-catenin into the nucleus (73). CagA-dependent activation of β-catenin mediated transcription of cancer-related genes had also been described in Mongolian gerbils and in patients infected with CagA-positive strains of *H. pylori* (81). Mongolian gerbils, apart from primates, are the only animal model available that develop gastric cancer upon *H. pylori* infection, without additional treatment with carcinogenic substances. In addition, CagA can induce the β-catenin pathway via activation of c-Met-associated PI3K-AKT signaling *in vitro* and *in vivo* (82). Further highlighting the importance of the β-catenin pathway is the finding that *H. pylori* can also induce β-catenin redistribution to the nucleus by additional CagA independent events (29). E-cadherin is also targeted by secreted HtrA protein of *H. pylori*, which is a serine protease that cleaves the ectodomain of E-cadherin further disrupting epithelial barrier function (83).

Thus, by disrupting tight-junctions via inhibition of Par-1b, adherence junctions via cadherin, and focal adhesions via activation of SHP-2, the CagA oncoprotein is potentially able to disrupt cell–cell and cell–matrix interaction of gastric epithelial cells. These are processes involved in EMT of cells.

### Epithelial to mesenchymal transition

Expression of CagA into polarized epithelial monolayer was found to be associated with transition of epithelial cells from a polarized state to an invasive phenotype, a cellular change characteristic of EMT (63). CagA-induced EMT depends on signaling triggered by the EPIYA motifs and localization of CagA to the junctions (63).

Epithelial to mesenchymal transition and mesenchymal–epithelial transitions (METs) have key roles in embryonic development, and their importance in the pathogenesis of cancer is increasingly recognized (84). EMT results from a complex molecular and cellular program by which epithelial cells de-differentiate loosing cell–cell adhesion and apical–basal polarity, and acquire mesenchymal features, including motility, invasiveness, and a heightened resistance to apoptosis. Similar to embryonic development, both EMT and MET seem to have crucial roles in the tumorigenic process. In particular, EMT has been found to contribute to invasion, metastatic dissemination, and acquisition of therapeutic resistance.

After the initial observation that CagA expression was able to induce a EMT-like process (63), the phenomenon has been studied in more detail and confirmed by other authors (85–87). Increased levels of the mesenchymal markers vimentin and fibronectin were detected in MDCK cells transfected with *cag*A (85). However, CagA expression did not down-regulate epithelial markers such as E-cadherin, α-catenin, β-catenin, and γ-catenin. Furthermore, there was no upregulation of EMT-inducing transcription factors, such as Twist and Snail, in cells expressing CagA. Therefore, CagA-expressing MDCK cells may undergo a peculiar EMT process in which both epithelial and mesenchymal markers are expressed simultaneously.

However, in another study in which three gastric epithelial cell lines (AGS, MGLVA1, and ST16) were co-cultured with *H. pylori*, upregulation of the EMT-associated genes Snail, Slug, and vimentin was observed (87). *H. pylori* also increased shedding of soluble heparin-binding epidermal growth factor (HB-EGF). Recent data suggest that soluble HB-EGF has a role in inducing EMT by upregulating EMT factors such as Slug (88, 89). This phenomenon was found to be partially dependent on both gastrin and matrix metalloproteinase (MMP)-7 expression. Indeed, inhibition of gastrin and MMP-7 expression through siRNAs, reduced upregulation of HB-EGF shedding and EMT gene expression. Interestingly, MMP-7 is a downstream transcriptional target of β-catenin following E-cadherin deregulation and has been linked to EMT and found upregulated in *H. pylori* infection.

Matrix metalloproteinase-7 is a member of a family of zinc-dependent proteolytic enzymes and is expressed and secreted primarily by well-differentiated epithelial cells. Increased levels of MMP-7 are present in many epithelial-derived malignancies, including gastric adenocarcinoma (90). Elevated levels of MMP-7 have also been detected in a high proportion of pre-malignant lesions in the stomach (gastric ulcers), suggesting that this protein plays an important role in early steps of the carcinogenic process. *H. pylori* was shown to increase MMP-7 expression in gastric epithelial cell lines in a cagPAI-dependent manner. This association was confirmed in gastric epithelial cells isolated from *H. pylori* infected patients. More recently, expression of MMP-7 was assessed by immunohistochemistry on 120 mucosal biopsies, of which 76 specimens with gastric epithelial dysplasia and 36 with intramucosal cancer (91). Greater expression of MMP-7 was confirmed in early-stage gastric cancer in association with cagPAI-positive strains.

Matrix metalloproteinases play an important role in controlling cell interactions with the ECM. MMPs are involved in the breakdown of ECM in normal physiological processes, such as embryonic development as well as in disease processes, such as cancer invasion and metastasis. However, an intriguing new hypothesis proposes that changes in ECM may play an active role during early stages of tumor formation *prior* to the onset of malignant invasion (92). Therefore, deregulation of cell–matrix interactions occurring during EMT would act as an epigenetic mechanism actively promoting cancer development. Indeed, disruption of cell–matrix interactions by ectopic expression of MMPs has been shown to be enough to induce carcinomas in animal models (93).

### Anti-apoptotic pathways

In an *H. pylori* experimental infection model in Mongolian gerbils, accumulation of the tumor suppressor factor p53 occurred at 4–6 h post-infection, followed by rapid decrease (94). Such a transient upregulation and downregulation of p53 was confirmed *in vitro*. This phenomenon was explained with the initial host response that up-regulates p53 expression, followed by CagA action that induces p53 degradation (94). Furthermore, *H. pylori* induces an apoptotic response of infected cells, which is inhibited by the delivery of CagA (95). These mechanisms have then been investigated *in vitro*, and it was demonstrated that CagA interacts with the tumor suppressor apoptosis-stimulating protein of p53-2 (ASPP2) (96). Upon DNA damage or oncogenic stimuli, ASPP2 binds and activates p53, inducing apoptosis. After interacting with CagA, ASPP2 is still able to bind p53, but then proteasomal degradation of p53 occurs, thus inhibiting the apoptotic response of the host cell (96). Thus, CagA is able to modulate the apoptotic signal that *H. pylori* itself induces. Even though the complete mechanism by which CagA hijacks and deregulates the tumor-suppression function of ASPP2 remains to be elucidated, the demonstration that CagA is involved in anti-apoptotic pathways is another important finding that confirms its strong relationship with EMT.

### STAT3 activation

Signal Transducer and Activator of Transcription 3 (STAT3) belongs to the STAT family of transcription factors and affects expression of cancer-related genes (97). *H. pylori* activates STAT3 via the IL-6/gp130 receptor in various cell lines and activation was dependent on CagA, but independent of CagA tyrosine phosphorylation (98). While a direct interaction of CagA with either IL-6 receptor or gp130 receptor could not been shown, the authors suggested that CagA triggers receptor heterodimerization by an indirect mechanism. The authors confirmed CagA-dependent STAT3 activation *in vivo* using the Mongolian gerbil model. Further evidence of the importance of STAT3 came from the study that demonstrated STAT3 activation in gastric tissue obtained from human subjects that were infected with CagA-positive strains. A study by Lee et al. also investigated CagA-dependent activation of the IL-6/gp130 receptor (99). Their finding confirmed the importance of CagA in STAT3 activation and additionally showed that the SHP-2/ERK was also induced following *Helicobacter*-mediated IL-6/gp130 receptor activation. Interestingly, in this study STAT3 activation was much stronger using a phosphorylation-resistant mutant of CagA than using wild type strains, while the opposite observation was made for activation of the SHP-2/ERK pathway. Thus, while CagA induces the ERK/MAP kinase pathway by direct interaction with and activation of SHP-2 (see above) it also up-regulates this pathway by indirect activation of the IL-6/gp130 receptor.

### Angiogenesis

The major characteristics of malignant cells are the following: deregulated cell proliferation, failure to differentiate, loss of normal apoptotic pathways, genetic instability, loss of replicative senescence, invasion, metastasis, evasion of the immune system, and increased angiogenesis (100). As described in the previous sections *H. pylori* affects most of these pathways and the following section summarizes the effects of *H. pylori* on angiogenesis.

Angiogenesis is defined as the physiological process through which new blood vessels form from pre-existing vessels (100). The newly formed endothelial cells (ECs) then migrate into the tumor and provide the condition for tumor growth and ultimately hematogenous spread. *H. pylori* appears to affect EC in different ways depending on the disease presentation. In peptic ulcer disease (PUD), presence of *H. pylori* was associated with delayed ulcer healing (101–104). In HUVEC cells water extracts of *H. pylori*-induced apoptosis independently of CagA or VacA (103), inhibited expression of angiogenic growth factor receptors (104) and triggered cytostasis of EC, likely by blocking the G1 to S phase cell-cycle transition (101, 102). Delayed wound healing and decreased proliferation together with increased epithelial damage caused by the infection may, in this scenario, cause chronic ulcer development. However, in gastritis and gastric cancer, opposite effects on angiogenesis do occur. Infection of the gastric mucosa with *H. pylori* causes a strong pro-inflammatory response, including activation of NF-kB and IL-8 (105). NF-kB induces expression of MMPs and angiogenic factors (106). Inflammation together with the effect of bacterial signaling factors may therefore act on EC in the vicinity of bacteria in the stomach and disturb their physiological function favoring tumor vascularization (107). Indeed, observations in patients with gastric adenocarcinoma have demonstrated a higher density of blood vessels in tumors before *H. pylori* eradication compared to after, suggesting a role of *H. pylori* in angiogenesis (108). *In vitro, H. pylori* also induced an increase in mRNA expression for IL-8, VEGF, angiogenin, urokinase-type plasminogen activator (uPA), and MMP-9 all of which are important mediators of angiogenic processes and gastric cell invasion (109, 110).

Cyclooxygenase 2 (COX-2) is an enzyme involved in prostaglandin biosynthesis and increase in COX-2 expression has been associated with various human cancers including colorectal, lung, pancreatic, esophageal, brain, and gastric cancers (111, 112). The cancer promoting functions of COX-2 may be explained by its ability to enhance cell proliferation, tumor cell invasion, and to induce angiogenesis (113). Indeed, COX-2 inhibitors reduce angiogenesis (114, 115). Chronic infection with *H. pylori* was shown to trigger upregulation of COX-2, which affects inflammatory processes and increased tissue damage (116). COX-2 expression was also upregulated in the human gastric mucosa of infected patients with gastric cancer suggesting that COX-2 upregulation plays a major role in gastric cancer development following infection.

### Effects on stem/progenitor cells

Although *H. pylori* and specifically CagA trigger many cancer-related signaling pathways *in vitro* and *in vivo*, many questions remain to be elucidated to fully understand the mechanisms behind the association of the pathogen with cancer. For example, it is not clear which cell type carries the potential for malignant transformation or which mechanisms trigger tumor initiation. The major cell type colonized with *H. pylori* is represented by the gastric pit or mucous-producing cells (117). Since this cell type is replaced too rapidly to allow the accumulation of mutations that promote transformation, recent research focuses on the potential role of long-lived gastric stem/progenitor cells localized in the isthmus region of the gland (118). The stem/progenitor cells are thought to make up a small fraction of all cancer cells (<1%). A model is emerging, in which *H. pylori* recruits and affects progenitor cells through chronic inflammation and cagPAI/CagA-induced oncogenic pathways. As a consequence the progenitor cells accumulate genetic and epigenetic modifications, which ultimately cause loss of homeostatic control and initiation of tumor development (119).

Research to characterize the interaction of *H. pylori* with progenitor cells is still in its infancy. However, *H. pylori* has been shown to interact with and invade epithelial progenitor cells (44). A gnotobiotic mouse model of chronic atrophic gastritis has been used to demonstrate that loss of parietal cells causes amplification of stem cells, which express sialylated receptors that can be used by *H. pylori* for adherence and cell invasion (120). A recent publication has demonstrated in a mouse model that *H. pylori* caused preneoplastic lesions, which contained bone marrow derived cells that were recruited to the gastric mucosa (121). The bone marrow derived cells are considered an alternate source of stem cells and likely are recruited along other stem cells to the isthmus region of the gland to produce the various types of gastric gland cells in the mucosa: pit, parietal, neck, and zymogenic cells (118, 122, 123).

These experiments indicate that CagA and possibly other *H. pylori* factors likely participate in the regulation of stem cell differentiation and contribute to the initiation of gastric cancer.

## Part 2 – *H. pylori* Epigenetic Mechanisms Affecting Gene Expression of Host Cell

Epigenetics has been defined as “the study of heritable changes in gene expression that occur independent of changes in the primary DNA sequence” (124). In other words, epigenetics studies the chromatin structure and its impact on gene function. Epigenetic modifications include DNA methylation, post-translational histone modification, nucleosome positioning along the DNA strand, and microRNA expression (125). These modifications are typically acquired during cell differentiation and control the accessibility of the genetic information by regulatory proteins (126).

Recent studies link epigenetic mechanisms to EMT that lead to oncogenesis (127, 128). Cancer cells have to acquire genetic as well as epigenetic changes to undergo through EMT and DNA methylation, histone modifications, and miRNAs appear to be associated with EMT and cancer progression.

### DNA methylation

DNA methylation occurs mainly on cytosine in repetitive CpG dinucleotides sequences, which are part of the majority of human promoter sequences (112, 129). Methylation of these CpG islands causes transcriptional silencing and furthermore may regulate active promoters (126). Various studies have reported that infection with *H. pylori* is associated with promoter methylation of various gastric cancer-associated genes (130–132) and eradication of the bacteria was able to reverse the process in patients with gastritis, but not in patients with intestinal metaplasia (133–135). Methylated genes included the O6-methylguanine DNA methyltransferase (DNA repair factor) (136), the trefoil factors 1 and 2 (regulators of gastric cell differentiation and proliferation) (137, 138), E-cadherin (133), GATA-4 and GATA-5 (134), p16 (cell-cycle control) (139), and IRX1 (cell-cycle control) (135). Another targeted promoter, the FOXD3 promoter, was identified using a genome-wide microarray-based approach, which compared methylation patterns of ca. 4500 CpG islands in mucosa samples of mice either infected or not infected with *H. pylori* (140). The study also compared mucosa of infected but asymptomatic individuals with mucosa of gastric cancer patients. FOXD3 is a member of the family of forkhead box transcriptional regulators. The FOXD3 promoter was found to be hypermethylated in both screens and progressively hypermethylated in more advanced lesions with the highest methylation level in human cancer cases. In agreement, FOXD3 was repressed in various gastric cancer cell lines and in more than 80% of gastric cancer cases. Furthermore, increased FOXD3 expression in cancer cell lines caused reduced proliferation rates, enhanced apoptosis, and reduced cell line invasiveness.

### Histone modifications

Chromatin is made of repeating units of nucleosomes, which contain DNA wrapped around an octamer of four histones proteins (H2a, H2b, H3, and H4). The N-terminus of histones can be post-translationally modified by methylation, acetylation, phosphorylation, ubiquitination, or sumoylation and the modification status affects DNA packing as well as gene transcription and DNA replication and repair (141). Nucleosome-free regions (NFRs) allow gene activation and transcription (142), while occlusion of the transcription start site with a nucleosome causes epigenetic gene silencing (143). Nucleosome remodeling has been closely linked to DNA methylation and histone modifications (144, 145).

Several recent reports have investigated the effects of *H. pylori* on histone modification. A chromatin immunoprecipitation analysis of NCI-N87 and primary gastric cells revealed that *H. pylori*-induced expression of the cell-cycle control factor p21(WAF)1. Induction followed the hyper-acetylation of histone H4 likely as a response to the release of HDAC-1 from the p21(WAF)1 promoter (146). HDAC-1 is a histone acetyltransferase that acetylates key lysine residues on H3 and H4 histones and acetylation activates transcription (147). *cag*PAI dependent dephosphorylation of histone H3 at serine 10 and threonine 3 was also shown, likely following transient pre-mitotic cell-cycle arrest and indeed, cell division cycle phosphatase CDC25C was strongly decreased during *H. pylori* infection (148). Similar effects on histone H3 phosphorylation were also reported by Ding et al., which furthermore demonstrated decreased acetylation of lysine 23 on histone H3 and this modification was associated with upregulation of the c-Jun proto-oncogene independent of the ERK/p38 pathways (149). Angrisano et al. demonstrated that *H. pylori* infection of gastric cells caused chromatin changes at the iNOS promoter. These changes included decreased methylation of lysine 9 on histone H3, but increased methylation and acetylation on histone H4, which were followed by increased iNOS expression (150). iNOS is the inducible nitric oxide synthase isoform, which is most commonly associated with malignant disease. Finally, *H. pylori* caused decreased expression of the gastric tumor suppressor protein p27 (151). Since p27 transcription was previously reported to be epigenetically regulated through histone acetylation via the G-protein coupled delta opioid receptor (DOR), histone acetylation, and acetyltransferase (p300) levels within the p27 promoter and DOR phosphorylation levels were measured. Infection of AGS and HS3C cells was associated with low p27 expression and reduced p27 promoter histone H4 acetylation. Recruitment of the p300 acetyltransferase and DOR phosphorylation were also decreased following infection with *H. pylori.*

### MicroRNA expression

miRNAs are small, non-coding RNAs that regulate gene expression through posttranscriptional gene silencing. They are typically 20–24 nucleotides long and pair with the 3′ untranslated regions of target messenger RNA to form the RNA-induced silencing complex (RISC). As a result of the complex formation the target RNA is then degraded or its translation inhibited (152). MicroRNAs are tissue specific and control many regulatory processes such as signal transduction, cell proliferation, apoptosis, angiogenesis, and differentiation and may also act as tumor suppressor genes (153). Considering such diverse regulatory functions it is not surprising that aberrant expression of miRNA has been linked to tumorogenesis (153) and various reports describe the role of miRNA in the development of gastric cancer specifically (154, 155). The effect of *H. pylori* on miRNA was recently comprehensively reviewed (156). It is becoming increasingly obvious that a considerable number of miRNAs is altered following infection with *H. pylori* and specific miRNA deregulation was found to contribute to host inflammation, cell-cycle progression, inhibition of apoptosis, cell invasion, and metastasis (156). One study of particular interest was using high throughput microarray screening to investigate the difference of miRNA signatures in *H. pylori* infected and uninfected gastric mucosa. The study found significant differences in the expression of 31 miRNAs and some of these, including the let-7 family members, required the expression of the cagPAI (157). Newer studies also pointed toward a role of the cagPAI and specifically CagA in regulating miRNA pathways. Zhu et al. demonstrated upregulation of miRNA-584 and miRNA-1290 in CagA-transformed cells and overexpression of both miRNAs induced intestinal metaplasia of gastric epithelial cells in knock-in mice (86). Interestingly, CagA-induced miRNA-584 and miRNA-1290 promote EMT through Foxa1, a critical factor in epithelial cell differentiation. These findings support a possible role of miRNA-584 and miRNA-1290 in deregulating cell differentiation and in promoting cancer through EMT. Saito et al. demonstrated that in polarized cells CagA-induced a mitogenic response via ERK activation. ERK prevented expression of p21(Waf1/Cip1) cyclin-dependent kinase inhibitor by activating c-Myc. c-Myc induced miRNA-17 and miRNA-20, which were both required to suppress p21(Waf1/Cip1). The opposite effect was observed in non-polarized cells, where upregulation of p21 (Waf1/Cip1) expression caused cell senescence (85). Interestingly, CagA was also shown to contribute to cell-cycle arrest in proliferating gastric tissue culture cells. CagA translocation caused a strong inhibition of miRNA-372 and miRNA-373, which both promote cell proliferation by silencing large tumor suppressor homolog 2 (LATS2) (158). The author suggested that this process might inhibit gastric epithelial renewal in favor of the colonizing bacteria. Future studies will be required to address these seemingly opposite effects of miRNAs during infection of the gastric epithelium with *H. pylori*.

## Part 3 – The Immune Response Against *H. pylori*: Role in Cancer Promotion and Against the Infection

The knowledge of the immune response to *H. pylori* is still incomplete: why the natural response seems to be ineffective and what is the protective response are questions that have been answered only in part. *H. pylori* infection elicits a strong immune response, at both B and T cell level. Nevertheless, the natural response seems unable to clear the infection, while the inflammatory response contributes to the pathology development, creating a microenvironment that may facilitate cellular transformation. Even early inflammatory events occurring upon *H. pylori* infection are relevant to the understanding of mechanisms behind malignant transformation. Indeed, atrophic gastritis, which is the most common and early outcome of *H. pylori* infection, leads to a significant increase in the risk of developing gastric cancer (159, 160).

*Helicobacter pylori* infection induces both innate effectors and a complex mix of Th1, Th17, and T regulatory cells (Treg) adaptive immune responses (161). Th1 response drives an inflammation that, if prolonged, results in pathological sequelae. On the other hand, experimental data showed that polarized Th2 response alone does not guarantee protection, suggesting that specific Th1 response appropriately tuned by Th2 cells would lead to a balanced, protective response (162–165). In the recent years, some advances in the knowledge of the contribution of both bacterial and host factors in determining the outcome of *H. pylori* infection have further filled some parts of the puzzle (166). To maintain colonization in the gastric tissue in spite of the robust immune response, *H. pylori* activates escaping mechanisms and exerts on the host immune system immunomodulatory action, through various factors (167, 168), establishing a relatively pacific coexistence. Nevertheless, the concomitance of certain host genetic background and particularly virulent *H. pylori* factors, such as CagA, can break this balance and lead to pathological outcomes including malignant lesions.

### Innate immune response: Pathogen-recognition receptors

Mammalian toll-like receptors (TLR) allow recognition of microbial molecules, with consequent initiation of the innate cellular responses against the invading pathogens. TLR2, TLR4, and TLR5 have been involved in the *H. pylori* recognition in the stomach (169–173), with bacterial factors such as CagA and HP-NAP modulating the interaction of the bacterium with TLR (169, 174), eventually leading to activation of nuclear factor-kappaB (NF-κB) and secretion of inflammatory cytokines. Interestingly, polymorphisms of these TLR have been reported to be associated with gastric carcinoma development (175, 176). However, TLR4 polymorphisms do not influence the risk of gastric cancer in Caucasian population (177). Also, association of gastric carcinogenesis with decreasing levels of TLR inhibitors and increased TLR2 and TLR4 levels has been reported (178), and chronic activation of TLR has been associated with tumor genesis (179).

The nucleotide-binding oligomerization domain (NOD) proteins are other important constituents of the innate immune response. NOD1 recognizes the bacterial peptidoglycan, resulting in signaling cascade that activates NF-κB and the production of pro-inflammatory cytokines (180). *H. pylori* activates NOD1 responses (181), dependent on *cag*PAI and its ability of delivering the bacterial peptidoglycan via the T4SS (182, 183). Also for NOD1, polymorphisms have been found to be associated with *H. pylori*-induced gastric mucosal inflammation (184), but not in the Caucasian population (177).

### Pro-inflammatory cytokines

*Helicobacter pylori* infection generates in the host a cytokine response that takes part in the disease development. Several studies indicate that polymorphisms of pro-inflammatory cytokines or of related genes may strongly influence the pathological outcome (181, 185, 186).

Some IL-1β and IL-1 receptor antagonist (IL-1RN) polymorphisms may increase the risk of malignant disease (187–190). This is conceivably linked to the suppression of gastric acid secretion induced by IL-1β. In a recent meta-analysis, ILRN2 polymorphism was found significantly associated with risk of gastric cancer in non-Asian populations, while reduced risk for Asian population was observed with IL-1β-31C polymorphism (191).

Polymorphisms of the human TNF-α (186, 192, 193), TNF-α promoter (194), and IL-10 (186, 195, 196) have been associated to higher risk of gastric cancer.

Polymorphisms in the Heat-shock protein 70 (HSP70)-1 have been reported to constitute possible risk factor for the development of precancerous lesions, gastric cancer, and duodenal ulcer (193). On the other hand, the BB genotype of HSP70-2 was found to be associated with a reduced risk of gastric pre-malignant condition in *H. pylori*-infected older individuals in the Japanese population (197). The effects of HSP70 can be due to the inhibitory activity exerted by HSP70 on IL-1 β and TNF-α production (198).

An IL-8 promoter polymorphism (IL-8-251A/T), which causes increased expression of IL-8, has been reported to be associated with progression of gastric atrophy in patients with *H. pylori* infection, thus increasing the risk of gastric ulcer and gastric cancer (199), as confirmed by a recent meta-analysis (200). The association of this IL-8 polymorphism with the risk of gastric cancer varies according to histological type, tumor location, *H. pylori* infection, and ethnicity/country (201, 202).

### T cell response

T-cell-mediated adaptive immunity is considered to play a major role in antitumor immunity (203). High density of tumor-infiltrating T cells (cytotoxic and memory in particular) was found associated with longer survival time of gastric cancer patients (204), and a specific T cell response of type I to cancer antigens, detectable in gastric cancer patients, has been proposed to have the potential of hampering tumor cell growth (203).

T regulatory (Treg) cells are a CD4^+^CD25^+^ population able to suppress the activation/proliferation of other T cells (205). Treg play a physiological role in protecting against autoimmune diseases suppressing T responses to self-antigens, and in controlling immune responses to pathogens (206). In *H. pylori*-infected subjects, suppression of the responsiveness of CD4^+^ memory cells has been observed, depending on the presence of *H. pylori*-specific Treg (207). Retrospective histopathological analysis performed on 167 subjects confirmed the local increase of Treg, finding an association between Treg, *H. pylori* infection, gastritis, peptic ulcer, and gastric adenocarcinoma (208). The Treg presence in the gastric mucosa of *H. pylori*-infected subjects suggested their involvement in suppressing mucosal immune responses, contributing to the infection persistence and modulating the *H. pylori*-induced gastritis (161, 209–211). With regard to gastric cancer, the accumulation of Treg in *H. pylori*-induced gastritis may prevent carcinogenesis, but in already established tumors they may promote tumor progression and metastasis (168).

A very recent study in a neonatal mouse model provided new insights on the role of Treg. Neonates responded to whole-cell *H. pylori* vaccination and were protected to an extent similar to that of the adults. The most intriguing results were observed in non-vaccinated animals: indeed, when infected with *H. pylori*, non-vaccinated neonates were protected from preneoplastic lesions, while adults were not (212), suggesting an active mechanism of peripheral tolerance induction. TGF-β was required for the development of tolerance. Depletion of Treg resulted in clearance of *H. pylori* accompanied by induction of gastric pathology (212). These observations indicate that tolerance can protect from severe disease outcomes, and may contribute to develop new gastric cancer prevention strategies.

A recent study observed that *H. pylori* infection induced a predominant Treg response in children, while the response was of Th17-type in adults (213). If on one hand this might account for the susceptibility of children to the infection and the lower degree of inflammatory cell infiltration observed in infected children (213), on the other hand it could further support the idea that immune response to *H. pylori* infection and the pathological outcome might be different depending on the age at which the colonization establishes.

Interestingly, CagA appears to be involved in mechanisms of T cell regulation. In mice, CagA-positive bacteria promoted the migration of *H. pylori*-primed CD4^+^ T cells to the site of infection, and CagA-dependent T cell priming elicited Treg-cell differentiation (214). Understanding of T cell response and of the mechanisms that *H. pylori* uses to shape the T cell response could be key to shedding new light on the mechanisms of *H. pylori* pathogenesis and to develop successful strategies against bacterial colonization and disease.

### Vaccines to prevent *H. pylori*-related cancer are still lacking

*Helicobacter pylori* infection in symptomatic subjects is generally treated. Current therapies include one proton pump inhibitor plus two antibiotics for 1–2 weeks. Eradication of *H. pylori* results in regression of gastric and duodenal ulcer as well as MALT lymphoma (215), and if performed before transformation process is too advanced, may prevent development of gastric cancer (216–218).

Unfortunately, the efficacy of the treatment has dropped below 80% (219, 220), mainly due to increasing antimicrobial resistance (clarithromycin in particular), but also to poor patient compliance with the multi-drug therapy. For this reason, several modifications in the combination and/or in the sequence of drug administration are under investigation (220–223). Although there are evidences that modified treatments and/or regimens are able in some cases to significantly increase the efficacy of the *H. pylori* eradication rates in comparison with the current standard therapy, antibiotic resistance remains a concern. Moreover, after a successful eradication, recurrence, or reinfection can occur (224).

Vaccination represents a valid alternative approach to overcome issues associated with reduced efficacy of antimicrobial-based therapies. An effective vaccine would prevent *H. pylori*-related diseases, including gastric cancer.

A large number of pre-clinical efficacy studies for vaccine candidates against *H. pylori* have been published, with promising results. However, a limited number of clinical trials were performed [reviewed in Ref. (225)]. In particular, several studies, conducted with urease-based vaccines, either administered as a purified recombinant protein along with mucosal adjuvants or as a *Salmonella*-vectored vaccine, showed limited immunogenicity and poor efficacy in humans. These disappointing results could discourage pharmaceutical companies to further invest in a *H. pylori* vaccine discovery and development. Nevertheless, in one of the latest studies with *Salmonella*-vectored vaccine, which included experimental challenge of volunteers, a small number of subjects cleared the infection, regardless of vaccination. In these subjects a T cell response to infection was observed that could be helpful in understanding mechanisms of protective response (226). Another vaccine, containing recombinant CagA, VacA, and HP-NAP proteins, was immunogenic and safe in a phase I clinical trial (227). No further results on clinical trials of *H. pylori* vaccines, and in particular of efficacy trials, were disclosed in the recent literature. Presently, there is not any anti-*H. pylori* vaccine licensed.

There are several reasons why currently the development of a *H. pylori* vaccine seems to be discontinued [exhaustively reviewed in Ref. (228)]. A major issue is the still incomplete knowledge of the mechanisms behind protective immunity against *H. pylori*. The majority of pre-clinical studies resulted in a significant decrease of bacterial colonization rather than complete, sterilizing, protection. This extent of efficacy against experimental infection in animals could be insufficient when translated to the human infection. Moreover, ideally a *H. pylori* vaccine would be both prophylactic and therapeutic, given the high rate of the currently infected population. Therefore, more research is needed to understand protective mechanisms and identify vaccine formulations able to prevent and cure the infection.

A further element that may limit the development of a *H. pylori* vaccine is represented by reports suggesting some beneficial roles of *H. pylori* colonization for the host (229, 230). Beneficial effects would be exerted in the first part of the host life, while detrimental effects start to appear over 50 years of age (229). Although it is clear that *H. pylori* infection can eventually lead to development of gastric cancer, the feeling that it could also provide the host with some advantages could adverse the compliance with a vaccination campaign.

In the frame of all these considerations, it could be now proposed for *H. pylori* vaccination an objective different than that of obtaining sterilizing immunity. Given that some of the most dangerous factors of *H. pylori* are already known, and that the relationships between CagA and *H. pylori*-induced carcinogenesis is well documented, it could be proposed a vaccine specifically targeting those factors. Such a vaccine should be aimed at affecting *H. pylori*-induced pathology rather than bacterial colonization. In other words, a still valuable *H. pylori* vaccine would be able to prevent gastric cancer, even without providing sterilizing immunity.

## Discussion

**Figure 2:**
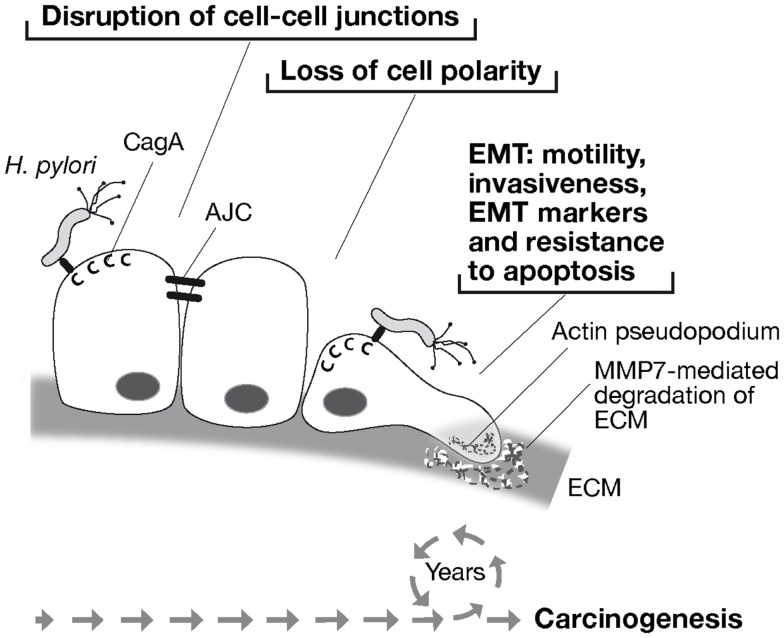
**Model of *H. pylori*-associated carcinogenesis through CagA-induced epithelial to mesenchymal transition (EMT)**. *H. pylori* injects CagA into gastric epithelial cells through a type IV secretion system. CagA disrupts cell–cell junctions by targeting the apical junction complex (AJC), causing loss of cell polarity. Thereafter, CagA induces cell motility and formation of actin pseudopodia, invasive behavior with the expression of the matrix metalloproteinase 7 (MMP-7), expression of EMT-associated genes, and resistance to apoptosis. Therefore, life-long exposure of the gastric mucosa to *H. pylori* and sustained injection of CagA into gastric epithelial cells may provide the epigenetic promoting forces toward carcinogenesis.

Unlike oncogenic retroviruses, *H. pylori* does not insert its genome into that of the host and CagA is a not an inheritable signal, so how can it contribute to carcinogenesis? The answer likely relies on the nature of *H. pylori* infection itself: it normally causes chronic infection persisting in the patient’s stomach virtually for his entire life. Therefore, the association between *H. pylori* and cancer might stem from the sustained injection of CagA and its ability to cause epithelial cell de-differentiation by disrupting cell–cell adhesion and apical–basal polarity, and acquire mesenchymal features, including motility, invasiveness, and a heightened resistance to apoptosis, in one word by inducing EMT (Figure 2).

Carcinogenesis is commonly discussed in terms of genetic alterations that lead to deregulation of cell growth. Recently, a role of epigenetic factors in promoting tumor initiation and progression by controlling cell–cell and cell–matrix interactions (92) as well as DNA methylation, histone modifications, and expression of micro RNAs (127) has been consolidated. By deregulating these processes, EMT may actively contribute to early stages of the carcinogenic process prior to malignant transformation (Figure 2).

Clearly, gastric cancer has a multifactorial etiology (185). On top of infection with *H. pylori*, and particularly with CagA-positive strains, contributing factors include diet and genetic background of the host. Variation of host immune response to the infection probably plays a key role in the disease outcome.

Current therapeutic approaches based on antibiotics, although are instrumental for curing *H. pylori* infected patients from most symptoms, present several limitations. Antibiotic resistance is raising and infection relapses are increasingly observed (219, 220). Eradication therapy is often administered to adults, who are generally thought to be exposed to the pathogen since their childhood. Indeed, infection normally occurs in infancy and persists for life (231). Therefore, prolonged exposure to the pathogen may cause irreversible damages in the patient. Over time the gastric mucosa infected with *H. pylori* undergoes through several changes. A model which describes the progression of gastric adenocarcinoma as consequence of *H. pylori* infection has been proposed (232). After the bacteria have colonized the gastric mucosa, over a period of few weeks, the infection causes acute gastritis. With the persistence of infection, gastritis can progress to atrophic, and during the years turn into intestinal metaplasia, dysplasia and, eventually, gastric adenocarcinoma.

Cancer progression may not be blocked by antibiotic therapy if it is done when pre-malignant lesions are already present (233–237). Unfortunately, no vaccines are commercially available as of today. If vaccines able to prevent and/or cure *H. pylori* infection would exist, they could substantially decrease the burden of gastric cancer. Indeed, vaccination would be the ideal approach to control *H. pylori* spread in the population. The success of such an approach has already been shown with vaccines against hepatitis B virus (HBV) in preventing liver cancer (238). Furthermore, reduction of the incidence of cervical cancer is expected with the use of human papillomavirus (HPV) vaccines, as reduction in precancerous lesions has been demonstrated in vaccinees (239).

Given the established association of CagA with cancer, a vaccine aimed at preventing this disease should most likely contain the toxin. However, further research on mechanisms of protection against the pathogen are needed in order to develop an effective vaccine. Given the lack of natural protection associated with *H. pylori* antibodies present in infected patients, it is likely that cellular immunity plays a major role. Therefore, the association of new generation adjuvants stimulating potent cellular response with the appropriate antibody response against key virulence factors (228), may represent the cornerstone of *H. pylori* vaccine development.

## Conflict of Interest Statement

The authors declare that the research was conducted in the absence of any commercial or financial relationships that could be construed as a potential conflict of interest.

## References

[1] Marshall BJ McGechie DB Francis GJ Utley PJ Pyloric *Campylobacter* serology. Lancet (1984) 2:281 10.1016/S0140-6736(84)90318-0 6146825

[2] Rothenbacher D Brenner H Burden of *Helicobacter pylori* and *H. pylori*-related diseases in developed countries: recent developments and future implications. Microbes Infect (2003) 5:693–703 10.1016/S1286-4579(03)00111-4 12814770

[3] Frenck RW Jr Clemens J *Helicobacter* in the developing world. Microbes Infect (2003) 5:705–13 10.1016/S1286-4579(03)00112-6 12814771

[4] Malaty HM El-Kasabany A Graham DY Miller CC Reddy SG Srinivasan SR Age at acquisition of *Helicobacter pylori* infection: a follow-up study from infancy to adulthood. Lancet (2002) 359:931–5 10.1016/S0140-6736(02)08025-X 11918912

[5] Rothenbacher D Winkler M Gonser T Adler G Brenner H Role of infected parents in transmission of *Helicobacter pylori* to their children. Pediatr Infect Dis J (2002) 21:674–9 10.1097/00006454-200207000-00014 12237602

[6] Parsonnet J Shmuely H Haggerty T Fecal and oral shedding of *Helicobacter pylori* from healthy infected adults. JAMA (1999) 282:2240–5 10.1001/jama.282.23.2240 10605976

[7] Zou QH Li RQ *Helicobacter pylori* in the oral cavity and gastric mucosa: a meta-analysis. J Oral Pathol Med (2011) 40:317–24 10.1111/j.1600-0714.2011.01006.x 21294774

[8] Lin HC Wu PY Friedman M Chang HW Wilson M Difference of *Helicobacter pylori* colonization in recurrent inflammatory and simple hyperplastic tonsil tissues. Arch Otolaryngol Head Neck Surg (2010) 136:468–70 10.1001/archoto.2010.63 20479377

[9] Vilarinho S Guimaraes NM Ferreira RM Gomes B Wen X Vieira MJ *Helicobacter pylori* colonization of the adenotonsillar tissue: fact or fiction? Int J Pediatr Otorhinolaryngol (2010) 74:807–11 10.1016/j.ijporl.2010.04.007 20452684

[10] Kusano K Inokuchi A Fujimoto K Miyamoto H Tokunaga O Kuratomi Y Coccoid *Helicobacter pylori* exists in the palatine tonsils of patients with IgA nephropathy. J Gastroenterol (2010) 45:406–12 10.1007/s00535-009-0169-9 19997853

[11] Goh KL Chan WK Shiota S Yamaoka Y Epidemiology of *Helicobacter pylori* infection and public health implications. Helicobacter (2011) 16(Suppl 1):1–9 10.1111/j.1523-5378.2011.00874.x 21896079 PMC3719046

[12] El-Omar EM Penman ID Ardill JE Chittajallu RS Howie C McColl KE *Helicobacter pylori* infection and abnormalities of acid secretion in patients with duodenal ulcer disease. Gastroenterology (1995) 109:681–91 10.1016/0016-5085(95)90374-7 7657096

[13] El-Omar EM Oien K El-Nujumi A Gillen D Wirz A Dahill S *Helicobacter pylori* infection and chronic gastric acid hyposecretion. Gastroenterology (1997) 113:15–24 10.1016/S0016-5085(97)70075-1 9207257

[14] Konturek PC Konturek SJ Brzozowski T *Helicobacter pylori* infection in gastric cancerogenesis. J Physiol Pharmacol (2009) 60:3–21 19826177

[15] Malfertheiner P The intriguing relationship of *Helicobacter pylori* infection and acid secretion in peptic ulcer disease and gastric cancer. Dig Dis (2011) 29:459–64 10.1159/000332213 22095010

[16] Herrera V Parsonnet J *Helicobacter pylori* and gastric adenocarcinoma. Clin Microbiol Infect (2009) 15:971–6 10.1111/j.1469-0691.2009.03031.x 19874380

[17] Crabtree JE Taylor JD Wyatt JI Heatley RV Shallcross TM Tompkins DS Mucosal IgA recognition of *Helicobacter pylori* 120 kDa protein, peptic ulceration, and gastric pathology. Lancet (1991) 338:332–5 10.1016/0140-6736(91)90477-7 1677696

[18] Ghiara P Marchetti M Blaser MJ Tummuru MK Cover TL Segal ED Role of the *Helicobacter pylori* virulence factors vacuolating cytotoxin, CagA, and urease in a mouse model of disease. Infect Immun (1995) 63:4154–60 7558333 10.1128/iai.63.10.4154-4160.1995 PMC173584

[19] Peek RM Jr Miller GG Tham KT Perez-Perez GI Zhao X Atherton JC Heightened inflammatory response and cytokine expression in vivo to cagA+ *Helicobacter pylori* strains. Lab Invest (1995) 73:760–70 8558837

[20] Covacci A Censini S Bugnoli M Petracca R Burroni D Macchia G Molecular characterization of the 128-kDa immunodominant antigen of *Helicobacter pylori* associated with cytotoxicity and duodenal ulcer. Proc Natl Acad Sci U S A (1993) 90:5791–5 10.1073/pnas.90.12.5791 8516329 PMC46808

[21] Censini S Lange C Xiang Z Crabtree JE Ghiara P Borodovsky M Cag, a pathogenicity island of *Helicobacter pylori*, encodes type I-specific and disease-associated virulence factors. Proc Natl Acad Sci U S A (1996) 93:14648–53 10.1073/pnas.93.25.14648 8962108 PMC26189

[22] Carrillo-Infante C Abbadessa G Bagella L Giordano A Viral infections as a cause of cancer (review). Int J Oncol (2007) 30:1521–8 17487374 10.3892/ijo.30.6.1521

[23] Han SU Kim YB Joo HJ Hahm KB Lee WH Cho YK *Helicobacter pylori* infection promotes gastric carcinogenesis in a mice model. J Gastroenterol Hepatol (2002) 17:253–61 10.1046/j.1440-1746.2002.02684.x 11982694

[24] Ogura K Maeda S Nakao M Watanabe T Tada M Kyutoku T Virulence factors of *Helicobacter pylori* responsible for gastric diseases in Mongolian gerbil. J Exp Med (2000) 192:1601–10 10.1084/jem.192.11.1601 11104802 PMC2193104

[25] Krueger S Roessner A Kuester D Murine models of *H. pylori*-induced gastritis and gastric adenocarcinoma. Pathol Res Pract (2011) 207:599–607 10.1016/j.prp.2011.09.005 22014883

[26] Ferlay J Shin HR Bray F Forman D Mathers C Parkin DM Estimates of worldwide burden of cancer in 2008: GLOBOCAN 2008. Int J Cancer (2010) 127:2893–917 10.1002/ijc.25516 21351269

[27] Wotherspoon AC Doglioni C Diss TC Pan L Moschini A de Boni M Regression of primary low-grade B-cell gastric lymphoma of mucosa-associated lymphoid tissue type after eradication of *Helicobacter pylori*. Lancet (1993) 342:575–7 10.1016/0140-6736(93)91409-F 8102719

[28] Hatakeyama M Linking epithelial polarity and carcinogenesis by multitasking *Helicobacter pylori* virulence factor CagA. Oncogene (2008) 27:7047–54 10.1038/onc.2008.353 19029944

[29] Polk DB Peek RM Jr *Helicobacter pylori*: gastric cancer and beyond. Nat Rev Cancer (2010) 10:403–14 10.1038/nrc2857 20495574 PMC2957472

[30] Ricci V Romano M Boquet P Molecular cross-talk between *Helicobacter pylori* and human gastric mucosa. World J Gastroenterol (2011) 17:1383–99 10.3748/wjg.v17.i11.1383 21472096 PMC3070011

[31] Covacci A Falkow S Berg DE Rappuoli R Did the inheritance of a pathogenicity island modify the virulence of *Helicobacter pylori*? Trends Microbiol (1997) 5:205–8 10.1016/S0966-842X(97)01035-4 9160510

[32] Akopyants NS Clifton SW Kersulyte D Crabtree JE Youree BE Reece CA Analyses of the cag pathogenicity island of *Helicobacter pylori*. Mol Microbiol (1998) 28:37–53 10.1046/j.1365-2958.1998.00770.x 9593295

[33] Yamaoka Y Mechanisms of disease: *Helicobacter pylori* virulence factors. Nat Rev Gastroenterol Hepatol (2010) 7:629–41 10.1038/nrgastro.2010.154 20938460 PMC3137895

[34] Cover TL Glupczynski Y Lage AP Burette A Tummuru MK Perez-Perez GI Serologic detection of infection with cagA+ *Helicobacter pylori* strains. J Clin Microbiol (1995) 33:1496–500 7650174 10.1128/jcm.33.6.1496-1500.1995 PMC228203

[35] Ohnishi N Yuasa H Tanaka S Sawa H Miura M Matsui A Transgenic expression of *Helicobacter pylori* CagA induces gastrointestinal and hematopoietic neoplasms in mouse. Proc Natl Acad Sci U S A (2008) 105:1003–8 10.1073/pnas.0711183105 18192401 PMC2242726

[36] Stein M Rappuoli R Covacci A Tyrosine phosphorylation of the *Helicobacter pylori* CagA antigen after cag-driven host cell translocation. Proc Natl Acad Sci U S A (2000) 97:1263–8 10.1073/pnas.97.3.1263 10655519 PMC15590

[37] Segal ED Cha J Lo J Falkow S Tompkins LS Altered states: involvement of phosphorylated CagA in the induction of host cellular growth changes by *Helicobacter pylori*. Proc Natl Acad Sci U S A (1999) 96:14559–64 10.1073/pnas.96.25.14559 10588744 PMC24475

[38] Odenbreit S Puls J Sedlmaier B Gerland E Fischer W Haas R Translocation of *Helicobacter pylori* CagA into gastric epithelial cells by type IV secretion. Science (2000) 287:1497–500 10.1126/science.287.5457.1497 10688800

[39] Asahi M Azuma T Ito S Ito Y Suto H Nagai Y *Helicobacter pylori* CagA protein can be tyrosine phosphorylated in gastric epithelial cells. J Exp Med (2000) 191:593–602 10.1084/jem.191.4.593 10684851 PMC2195829

[40] Conradi J Tegtmeyer N Wozna M Wissbrock M Michalek C Gagell C An RGD helper sequence in CagL of *Helicobacter pylori* assists in interactions with integrins and injection of CagA. Front Cell Infect Microbiol (2012) 2:70 10.3389/fcimb.2012.00070 22919661 PMC3417467

[41] Kwok T Zabler D Urman S Rohde M Hartig R Wessler S *Helicobacter* exploits integrin for type IV secretion and kinase activation. Nature (2007) 449:862–6 10.1038/nature06187 17943123

[42] Jimenez-Soto LF Kutter S Sewald X Ertl C Weiss E Kapp U *Helicobacter pylori* type IV secretion apparatus exploits beta1 integrin in a novel RGD-independent manner. PLoS Pathog (2009) 5:e1000684 10.1371/journal.ppat.1000684 19997503 PMC2779590

[43] Kaplan-Turkoz B Jimenez-Soto LF Dian C Ertl C Remaut H Louche A Structural insights into *Helicobacter pylori* oncoprotein CagA interaction with beta1 integrin. Proc Natl Acad Sci U S A (2012) 109:14640–5 10.1073/pnas.1206098109 22908298 PMC3437852

[44] Necchi V Candusso ME Tava F Luinetti O Ventura U Fiocca R Intracellular, intercellular, and stromal invasion of gastric mucosa, preneoplastic lesions, and cancer by *Helicobacter pylori*. Gastroenterology (2007) 132:1009–23 10.1053/j.gastro.2007.01.049 17383424

[45] Murata-Kamiya N Kikuchi K Hayashi T Higashi H Hatakeyama M *Helicobacter pylori* exploits host membrane phosphatidylserine for delivery, localization, and pathophysiological action of the CagA oncoprotein. Cell Host Microbe (2010) 7:399–411 10.1016/j.chom.2010.04.005 20478541

[46] Hayashi T Senda M Morohashi H Higashi H Horio M Kashiba Y Tertiary structure-function analysis reveals the pathogenic signaling potentiation mechanism of *Helicobacter pylori* oncogenic effector CagA. Cell Host Microbe (2012) 12:20–33 10.1016/j.chom.2012.05.010 22817985

[47] Stein M Bagnoli F Halenbeck R Rappuoli R Fantl WJ Covacci A c-Src/Lyn kinases activate *Helicobacter pylori* CagA through tyrosine phosphorylation of the EPIYA motifs. Mol Microbiol (2002) 43:971–80 10.1046/j.1365-2958.2002.02781.x 11929545

[48] Tegtmeyer N Backert S Role of Abl and Src family kinases in actin-cytoskeletal rearrangements induced by the *Helicobacter pylori* CagA protein. Eur J Cell Biol (2011) 90:880–90 10.1016/j.ejcb.2010.11.006 21247656

[49] Higashi H Tsutsumi R Muto S Sugiyama T Azuma T Asaka M SHP-2 tyrosine phosphatase as an intracellular target of *Helicobacter pylori* CagA protein. Science (2002) 295:683–6 10.1126/science.1067147 11743164

[50] Neel BG Gu H Pao L The ‘Shp’ing news: SH2 domain-containing tyrosine phosphatases in cell signaling. Trends Biochem Sci (2003) 28:284–93 10.1016/S0968-0004(03)00091-4 12826400

[51] Hatakeyama M Oncogenic mechanisms of the *Helicobacter pylori* CagA protein. Nat Rev Cancer (2004) 4:688–94 10.1038/nrc1433 15343275

[52] Felici A Giubellino A Bottaro DP Gab1 mediates hepatocyte growth factor-stimulated mitogenicity and morphogenesis in multipotent myeloid cells. J Cell Biochem (2010) 111:310–21 10.1002/jcb.22695 20506405 PMC3393599

[53] Tsutsumi R Takahashi A Azuma T Higashi H Hatakeyama M Focal adhesion kinase is a substrate and downstream effector of SHP-2 complexed with *Helicobacter pylori* CagA. Mol Cell Biol (2006) 26:261–76 10.1128/MCB.26.1.261-276.2006 16354697 PMC1317644

[54] Tsutsumi R Higashi H Higuchi M Okada M Hatakeyama M Attenuation of *Helicobacter pylori* CagA x SHP-2 signaling by interaction between CagA and C-terminal Src kinase. J Biol Chem (2003) 278:3664–70 10.1074/jbc.M208155200 12446738

[55] Mimuro H Suzuki T Tanaka J Asahi M Haas R Sasakawa C Grb2 is a key mediator of *Helicobacter pylori* CagA protein activities. Mol Cell (2002) 10:745–55 10.1016/S1097-2765(02)00681-0 12419219

[56] Suzuki M Mimuro H Suzuki T Park M Yamamoto T Sasakawa C Interaction of CagA with Crk plays an important role in *Helicobacter pylori*-induced loss of gastric epithelial cell adhesion. J Exp Med (2005) 202:1235–47 10.1084/jem.20051027 16275761 PMC2213224

[57] Churin Y Al-Ghoul L Kepp O Meyer TF Birchmeier W Naumann M *Helicobacter pylori* CagA protein targets the c-Met receptor and enhances the motogenic response. J Cell Biol (2003) 161:249–55 10.1083/jcb.200208039 12719469 PMC2172921

[58] Selbach M Moese S Hurwitz R Hauck CR Meyer TF Backert S The *Helicobacter pylori* CagA protein induces cortactin dephosphorylation and actin rearrangement by c-Src inactivation. EMBO J (2003) 22:515–28 10.1093/emboj/cdg050 12554652 PMC140734

[59] Hazell SL Lee A Brady L Hennessy W *Campylobacter pyloridis* and gastritis: association with intercellular spaces and adaptation to an environment of mucus as important factors in colonization of the gastric epithelium. J Infect Dis (1986) 153:658–63 10.1093/infdis/153.4.658 3950447

[60] Steer H Mucosa-related bacteria in the stomach. Lancet (1984) 2:528 10.1016/S0140-6736(84)92612-6 6147592

[61] Noach LA Rolf TM Tytgat GN Electron microscopic study of association between *Helicobacter pylori* and gastric and duodenal mucosa. J Clin Pathol (1994) 47:699–704 10.1136/jcp.47.8.699 7962619 PMC502139

[62] Amieva MR Vogelmann R Covacci A Tompkins LS Nelson WJ Falkow S Disruption of the epithelial apical-junctional complex by *Helicobacter pylori* CagA. Science (2003) 300:1430–4 10.1126/science.1081919 12775840 PMC3369828

[63] Bagnoli F Buti L Tompkins L Covacci A Amieva MR *Helicobacter pylori* CagA induces a transition from polarized to invasive phenotypes in MDCK cells. Proc Natl Acad Sci U S A (2005) 102:16339–44 10.1073/pnas.0502598102 16258069 PMC1274241

[64] Zeaiter Z Cohen D Musch A Bagnoli F Covacci A Stein M Analysis of detergent-resistant membranes of *Helicobacter pylori* infected gastric adenocarcinoma cells reveals a role for MARK2/Par1b in CagA-mediated disruption of cellular polarity. Cell Microbiol (2008) 10:781–94 10.1111/j.1462-5822.2007.01084.x 18005242

[65] Tan S Tompkins LS Amieva MR *Helicobacter pylori* usurps cell polarity to turn the cell surface into a replicative niche. PLoS Pathog (2009) 5:e1000407 10.1371/journal.ppat.1000407 19412339 PMC2669173

[66] Saadat I Higashi H Obuse C Umeda M Murata-Kamiya N Saito Y *Helicobacter pylori* CagA targets PAR1/MARK kinase to disrupt epithelial cell polarity. Nature (2007) 447:330–3 10.1038/nature05765 17507984

[67] Drewes G Ebneth A Preuss U Mandelkow EM Mandelkow E MARK, a novel family of protein kinases that phosphorylate microtubule-associated proteins and trigger microtubule disruption. Cell (1997) 89:297–308 10.1016/S0092-8674(00)80208-1 9108484

[68] Bohm H Brinkmann V Drab M Henske A Kurzchalia TV Mammalian homologues of *C. elegans* PAR-1 are asymmetrically localized in epithelial cells and may influence their polarity. Curr Biol (1997) 7:603–6 10.1016/S0960-9822(06)00260-0 9259552

[69] Cohen D Brennwald PJ Rodriguez-Boulan E Musch A Mammalian PAR-1 determines epithelial lumen polarity by organizing the microtubule cytoskeleton. J Cell Biol (2004) 164:717–27 10.1083/jcb.200308104 14981097 PMC2172160

[70] Nesic D Miller MC Quinkert ZT Stein M Chait BT Stebbins CE *Helicobacter pylori* CagA inhibits PAR1-MARK family kinases by mimicking host substrates. Nat Struct Mol Biol (2010) 17:130–2 10.1038/nsmb.1705 19966800 PMC3006182

[71] Yamahashi Y Hatakeyama M PAR1b takes the stage in the morphogenetic and motogenetic activity of *Helicobacter pylori* CagA oncoprotein. Cell Adh Migr (2013) 7:11–8 10.4161/cam.21936 23076215 PMC3544774

[72] Cheng IK Tsang BC Lai KP Ching AK Chan AW To KF GEF-H1 over-expression in hepatocellular carcinoma promotes cell motility via activation of RhoA signalling. J Pathol (2012) 228(4):575–85 10.1002/path.4084 22847784

[73] Murata-Kamiya N Kurashima Y Teishikata Y Yamahashi Y Saito Y Higashi H *Helicobacter pylori* CagA interacts with E-cadherin and deregulates the beta-catenin signal that promotes intestinal transdifferentiation in gastric epithelial cells. Oncogene (2007) 26:4617–26 10.1038/sj.onc.1210251 17237808

[74] Kurashima Y Murata-Kamiya N Kikuchi K Higashi H Azuma T Kondo S Deregulation of beta-catenin signal by *Helicobacter pylori* CagA requires the CagA-multimerization sequence. Int J Cancer (2008) 122:823–31 10.1002/ijc.23190 17960618

[75] Oliveira MJ Costa AM Costa AC Ferreira RM Sampaio P Machado JC CagA associates with c-Met, E-cadherin, and p120-catenin in a multiproteic complex that suppresses *Helicobacter pylori*-induced cell-invasive phenotype. J Infect Dis (2009) 200:745–55 10.1086/604727 19604117

[76] Tian X Liu Z Niu B Zhang J Tan TK Lee SR E-cadherin/beta-catenin complex and the epithelial barrier. J Biomed Biotechnol (2011) 2011:567305 10.1155/2011/567305 22007144 PMC3191826

[77] Carneiro P Fernandes MS Figueiredo J Caldeira J Carvalho J Pinheiro H E-cadherin dysfunction in gastric cancer – cellular consequences, clinical applications and open questions. FEBS Lett (2012) 586:2981–9 10.1016/j.febslet.2012.07.045 22841718

[78] Guilford P Hopkins J Harraway J McLeod M McLeod N Harawira P E-cadherin germline mutations in familial gastric cancer. Nature (1998) 392:402–5 10.1038/32918 9537325

[79] Kikuchi A Yamamoto H Sato A Matsumoto S New insights into the mechanism of Wnt signaling pathway activation. Int Rev Cell Mol Biol (2011) 291:21–71 10.1016/B978-0-12-386035-4.00002-1 22017973

[80] Niehrs C The complex world of WNT receptor signalling. Nat Rev Mol Cell Biol (2012) 13:767–79 10.1038/nrm3470 23151663

[81] Franco AT Israel DA Washington MK Krishna U Fox JG Rogers AB Activation of beta-catenin by carcinogenic *Helicobacter pylori*. Proc Natl Acad Sci U S A (2005) 102:10646–51 10.1073/pnas.0504927102 16027366 PMC1180811

[82] Suzuki M Mimuro H Kiga K Fukumatsu M Ishijima N Morikawa H *Helicobacter pylori* CagA phosphorylation-independent function in epithelial proliferation and inflammation. Cell Host Microbe (2009) 5:23–34 10.1016/j.chom.2008.11.010 19154985

[83] Hoy B Lower M Weydig C Carra G Tegtmeyer N Geppert T *Helicobacter pylori* HtrA is a new secreted virulence factor that cleaves E-cadherin to disrupt intercellular adhesion. EMBO Rep (2010) 11:798–804 10.1038/embor.2010.114 20814423 PMC2948180

[84] Polyak K Weinberg RA Transitions between epithelial and mesenchymal states: acquisition of malignant and stem cell traits. Nat Rev Cancer (2009) 9:265–73 10.1038/nrc2620 19262571

[85] Saito Y Murata-Kamiya N Hirayama T Ohba Y Hatakeyama M Conversion of *Helicobacter pylori* CagA from senescence inducer to oncogenic driver through polarity-dependent regulation of p21. J Exp Med (2010) 207:2157–74 10.1084/jem.20100602 20855497 PMC2947069

[86] Zhu Y Jiang Q Lou X Ji X Wen Z Wu J MicroRNAs up-regulated by CagA of *Helicobacter pylori* induce intestinal metaplasia of gastric epithelial cells. PLoS One (2012) 7:e35147 10.1371/journal.pone.0035147 22536353 PMC3335061

[87] Yin Y Grabowska AM Clarke PA Whelband E Robinson K Argent RH *Helicobacter pylori* potentiates epithelial:mesenchymal transition in gastric cancer: links to soluble HB-EGF, gastrin and matrix metalloproteinase-7. Gut (2010) 59:1037–45 10.1136/gut.2009.199794 20584780 PMC2976077

[88] Smith JP Pozzi A Dhawan P Singh AB Harris RC Soluble HB-EGF induces epithelial-to-mesenchymal transition in inner medullary collecting duct cells by upregulating Snail-2. Am J Physiol Renal Physiol (2009) 296:F957–65 10.1152/ajprenal.90490.2008 19244405 PMC2681361

[89] Yagi H Yotsumoto F Miyamoto S Heparin-binding epidermal growth factor-like growth factor promotes transcoelomic metastasis in ovarian cancer through epithelial-mesenchymal transition. Mol Cancer Ther (2008) 7:3441–51 10.1158/1535-7163.MCT-08-0417 18852147

[90] Ii M Yamamoto H Adachi Y Maruyama Y Shinomura Y Role of matrix metalloproteinase-7 (matrilysin) in human cancer invasion, apoptosis, growth, and angiogenesis. Exp Biol Med (Maywood) (2006) 231:20–7 16380641 10.1177/153537020623100103

[91] Chung WC Jung SH Lee KM Paik CN Kawk JW Jung JH The detection of *Helicobacter pylori* cag pathogenicity islands (PAIs) and expression of matrix metalloproteinase-7 (MMP-7) in gastric epithelial dysplasia and intramucosal cancer. Gastric Cancer (2010) 13:162–9 10.1007/s10120-010-0552-5 20820985

[92] Ingber DE Cancer as a disease of epithelial-mesenchymal interactions and extracellular matrix regulation. Differentiation (2002) 70:547–60 10.1046/j.1432-0436.2002.700908.x 12492496

[93] Sternlicht MD Lochter A Sympson CJ Huey B Rougier JP Gray JW The stromal proteinase MMP3/stromelysin-1 promotes mammary carcinogenesis. Cell (1999) 98:137–46 10.1016/S0092-8674(00)81009-0 10428026 PMC2853255

[94] Wei J Nagy TA Vilgelm A Zaika E Ogden SR Romero-Gallo J Regulation of p53 tumor suppressor by *Helicobacter pylori* in gastric epithelial cells. Gastroenterology (2010) 139:1333–43 10.1053/j.gastro.2010.06.018 20547161 PMC2949494

[95] Mimuro H Suzuki T Nagai S Rieder G Suzuki M Nagai T *Helicobacter pylori* dampens gut epithelial self-renewal by inhibiting apoptosis, a bacterial strategy to enhance colonization of the stomach. Cell Host Microbe (2007) 2:250–63 10.1016/j.chom.2007.09.005 18005743

[96] Buti L Spooner E Van der Veen AG Rappuoli R Covacci A Ploegh HL *Helicobacter pylori* cytotoxin-associated gene A (CagA) subverts the apoptosis-stimulating protein of p53 (ASPP2) tumor suppressor pathway of the host. Proc Natl Acad Sci U S A (2011) 108:9238–43 10.1073/pnas.1106200108 21562218 PMC3107298

[97] Huang S Regulation of metastases by signal transducer and activator of transcription 3 signaling pathway: clinical implications. Clin Cancer Res (2007) 13:1362–6 10.1158/1078-0432.CCR-06-2313 17332277

[98] Bronte-Tinkew DM Terebiznik M Franco A Ang M Ahn D Mimuro H *Helicobacter pylori* cytotoxin-associated gene A activates the signal transducer and activator of transcription 3 pathway in vitro and in vivo. Cancer Res (2009) 69:632–9 10.1158/0008-5472.CAN-08-1191 19147578 PMC3418672

[99] Lee IO Kim JH Choi YJ Pillinger MH Kim SY Blaser MJ *Helicobacter pylori* CagA phosphorylation status determines the gp130-activated SHP2/ERK and JAK/STAT signal transduction pathways in gastric epithelial cells. J Biol Chem (2010) 285:16042–50 10.1074/jbc.M110.111054 20348091 PMC2871473

[100] Hanahan D Weinberg RA Hallmarks of cancer: the next generation. Cell (2011) 144:646–74 10.1016/j.cell.2011.02.013 21376230

[101] Jenkinson L Bardhan KD Atherton J Kalia N *Helicobacter pylori* prevents proliferative stage of angiogenesis in vitro: role of cytokines. Dig Dis Sci (2002) 47:1857–62 10.1023/A:1016469217449 12184542

[102] Pearce HR Kalia N Bardhan KD Atherton JC Brown NJ Effects of *Helicobacter pylori* on endothelial cell proliferation and chemotaxis. Digestion (2004) 69:201–10 10.1159/000079149 15205568

[103] Kurosawa A Miwa H Hirose M Tsune I Nagahara A Sato N Inhibition of cell proliferation and induction of apoptosis by *Helicobacter pylori* through increased phosphorylated p53, p21 and Bax expression in endothelial cells. J Med Microbiol (2002) 51:385–91 11990490 10.1099/0022-1317-51-5-385

[104] Kim JS Kim JM Jung HC Song IS *Helicobacter pylori* down-regulates the receptors of vascular endothelial growth factor and angiopoietin in vascular endothelial cells: implications in the impairment of gastric ulcer healing. Dig Dis Sci (2004) 49:778–86 10.1023/B:DDAS.0000030089.76514.e4 15259499

[105] Sharma SA Tummuru MK Blaser MJ Kerr LD Activation of IL-8 gene expression by *Helicobacter pylori* is regulated by transcription factor nuclear factor-kappa B in gastric epithelial cells. J Immunol (1998) 160:2401–7 9498783

[106] Lamb A Chen LF Role of the *Helicobacter pylori*-induced inflammatory response in the development of gastric cancer. J Cell Biochem (2013) 114:491–7 10.1002/jcb.24389 22961880 PMC3909030

[107] Kalia N Jacob S Brown NJ Reed MW Morton D Bardhan KD Studies on the gastric mucosal microcirculation. 2. *Helicobacter pylori* water soluble extracts induce platelet aggregation in the gastric mucosal microcirculation in vivo. Gut (1997) 41:748–52 10.1136/gut.41.6.748 9462206 PMC1891607

[108] Sasaki A Kitadai Y Ito M Sumii M Tanaka S Yoshihara M *Helicobacter pylori* infection influences tumor growth of human gastric carcinomas. Scand J Gastroenterol (2003) 38:153–8 10.1080/00365520310000636 12678331

[109] Kitadai Y Sasaki A Ito M Tanaka S Oue N Yasui W *Helicobacter pylori* infection influences expression of genes related to angiogenesis and invasion in human gastric carcinoma cells. Biochem Biophys Res Commun (2003) 311:809–14 10.1016/j.bbrc.2003.10.077 14623253

[110] Strowski MZ Cramer T Schafer G Juttner S Walduck A Schipani E *Helicobacter pylori* stimulates host vascular endothelial growth factor-A (vegf-A) gene expression via MEK/ERK-dependent activation of Sp1 and Sp3. FASEB J (2004) 18:218–20 14597566 10.1096/fj.03-0055fje

[111] Dannenberg AJ Altorki NK Boyle JO Dang C Howe LR Weksler BB Cyclo-oxygenase 2: a pharmacological target for the prevention of cancer. Lancet Oncol (2001) 2:544–51 10.1016/S1470-2045(01)00488-0 11905709

[112] Aaltonen T Abulencia A Adelman J Affolder T Akimoto T Albrow MG Search for exclusive gammagamma production in Hadron-Hadron collisions. Phys Rev Lett (2007) 99:242002 10.1103/PhysRevLett.99.242002 18233441

[113] Salvado MD Alfranca A Haeggstrom JZ Redondo JM Prostanoids in tumor angiogenesis: therapeutic intervention beyond COX-2. Trends Mol Med (2012) 18:233–43 10.1016/j.molmed.2012.02.002 22425675

[114] Masferrer JL Leahy KM Koki AT Zweifel BS Settle SL Woerner BM Antiangiogenic and antitumor activities of cyclooxygenase-2 inhibitors. Cancer Res (2000) 60:1306–11 10728691

[115] Leahy KM Ornberg RL Wang Y Zweifel BS Koki AT Masferrer JL Cyclooxygenase-2 inhibition by celecoxib reduces proliferation and induces apoptosis in angiogenic endothelial cells in vivo. Cancer Res (2002) 62:625–31 11830509

[116] Zarrilli R Tuccillo C Santangelo M Nardone G Romano M Increased COX-2, but not COX-1, mRNA expression in *Helicobacter pylori* gastritis. Am J Gastroenterol (1999) 94:3376–8 10.1111/j.1572-0241.1999.03376.x 10566756

[117] Mueller A Merrell DS Grimm J Falkow S Profiling of microdissected gastric epithelial cells reveals a cell type-specific response to *Helicobacter pylori* infection. Gastroenterology (2004) 127:1446–62 10.1053/j.gastro.2004.08.054 15521014

[118] Karam SM Cellular origin of gastric cancer. Ann N Y Acad Sci (2008) 1138:162–8 10.1196/annals.1414.023 18837897

[119] Ding SZ Zheng PY *Helicobacter pylori* infection induced gastric cancer; advance in gastric stem cell research and the remaining challenges. Gut Pathog (2012) 4:18 10.1186/1757-4749-4-18 23217022 PMC3536631

[120] Oh JD Karam SM Gordon JI Intracellular *Helicobacter pylori* in gastric epithelial progenitors. Proc Natl Acad Sci U S A (2005) 102:5186–91 10.1073/pnas.0407657102 15795379 PMC555607

[121] Varon C Dubus P Mazurier F Asencio C Chambonnier L Ferrand J *Helicobacter pylori* infection recruits bone marrow-derived cells that participate in gastric preneoplasia in mice. Gastroenterology (2012) 142:281–91 10.1053/j.gastro.2011.10.036 22062361

[122] Okumura T Wang SS Takaishi S Tu SP Ng V Ericksen RE Identification of a bone marrow-derived mesenchymal progenitor cell subset that can contribute to the gastric epithelium. Lab Invest (2009) 89:1410–22 10.1038/labinvest.2009.88 19841619 PMC2917339

[123] Houghton J Stoicov C Nomura S Rogers AB Carlson J Li H Gastric cancer originating from bone marrow-derived cells. Science (2004) 306:1568–71 10.1126/science.1099513 15567866

[124] Waddington CH Canalization of development and the inheritance of acquired characters. Nature (1942) 150:563–65 10.1038/150563a0 13666847

[125] Jones PA Baylin SB The epigenomics of cancer. Cell (2007) 128:683–92 10.1016/j.cell.2007.01.029 17320506 PMC3894624

[126] Suzuki MM Bird A DNA methylation landscapes: provocative insights from epigenomics. Nat Rev Genet (2008) 9:465–76 10.1038/nrg2341 18463664

[127] Stadler SC Allis CD Linking epithelial-to-mesenchymal-transition and epigenetic modifications. Semin Cancer Biol (2012) 22:404–10 10.1016/j.semcancer.2012.06.007 22706095 PMC3445725

[128] Wang Y Li W Zang X Chen N Liu T Tsonis PA MicroRNA-204-5p regulates epithelial-to-mesenchymal transition during human posterior capsule opacification by targeting SMAD4. Invest Ophthalmol Vis Sci (2013) 54:323–32 10.1167/iovs.12-10904 23221074

[129] Bird A DNA methylation patterns and epigenetic memory. Genes Dev (2002) 16:6–21 10.1101/gad.947102 11782440

[130] Maekita T Nakazawa K Mihara M Nakajima T Yanaoka K Iguchi M High levels of aberrant DNA methylation in *Helicobacter pylori*-infected gastric mucosae and its possible association with gastric cancer risk. Clin Cancer Res (2006) 12:989–95 10.1158/1078-0432.CCR-05-2096 16467114

[131] Nardone G Compare D De Colibus P de Nucci G Rocco A *Helicobacter pylori* and epigenetic mechanisms underlying gastric carcinogenesis. Dig Dis (2007) 25:225–9 10.1159/000103890 17827945

[132] Yoshida T Kato J Maekita T Yamashita S Enomoto S Ando T Altered mucosal DNA methylation in parallel with highly active *Helicobacter pylori*-related gastritis. Gastric Cancer (2013). 10.1007/s10120-012-0230-x 23292007

[133] Chan AO Peng JZ Lam SK Lai KC Yuen MF Cheung HK Eradication of *Helicobacter pylori* infection reverses E-cadherin promoter hypermethylation. Gut (2006) 55:463–8 10.1136/gut.2005.077776 16428266 PMC1856151

[134] Wen XZ Akiyama Y Pan KF Liu ZJ Lu ZM Zhou J Methylation of GATA-4 and GATA-5 and development of sporadic gastric carcinomas. World J Gastroenterol (2010) 16:1201–8 10.3748/wjg.v16.i10.1201 20222162 PMC2839171

[135] Guo XB Guo L Zhi QM Ji J Jiang JL Zhang RJ *Helicobacter pylori* induces promoter hypermethylation and downregulates gene expression of IRX1 transcription factor on human gastric mucosa. J Gastroenterol Hepatol (2011) 26:1685–90 10.1111/j.1440-1746.2011.06808.x 21649733

[136] Sepulveda AR Yao Y Yan W Park DI Kim JJ Gooding W CpG methylation and reduced expression of O6-methylguanine DNA methyltransferase is associated with *Helicobacter pylori* infection. Gastroenterology (2010) 138:1836–44 10.1053/j.gastro.2009.12.042 20044995

[137] Tomita H Takaishi S Menheniott TR Yang X Shibata W Jin G Inhibition of gastric carcinogenesis by the hormone gastrin is mediated by suppression of TFF1 epigenetic silencing. Gastroenterology (2011) 140:879–91 10.1053/j.gastro.2010.11.037 21111741 PMC3049860

[138] Peterson AJ Menheniott TR O’Connor L Walduck AK Fox JG Kawakami K *Helicobacter pylori* infection promotes methylation and silencing of trefoil factor 2, leading to gastric tumor development in mice and humans. Gastroenterology (2010) 139:2005–17 10.1053/j.gastro.2010.08.043 20801119 PMC3970568

[139] Park S Kim KM Kim JJ Lee JH Rhee JC Ko YH Methylation of p16INK4A and mitotic arrest defective protein 2 (MAD2) genes in gastric marginal-zone B-cell lymphomas. Acta Haematol (2008) 120:217–24 10.1159/000195698 19174606

[140] Cheng AS Li MS Kang W Cheng VY Chou JL Lau SS *Helicobacter pylori* causes epigenetic dysregulation of FOXD3 to promote gastric carcinogenesis. Gastroenterology (2013) 144(2–133):e9 10.1053/j.gastro.2012.10.002 23058321

[141] Kouzarides T Chromatin modifications and their function. Cell (2007) 128:693–705 10.1016/j.cell.2007.02.005 17320507

[142] Lin JC Jeong S Liang G Takai D Fatemi M Tsai YC Role of nucleosomal occupancy in the epigenetic silencing of the MLH1 CpG island. Cancer Cell (2007) 12:432–44 10.1016/j.ccr.2007.10.014 17996647 PMC4657456

[143] Schones DE Cui K Cuddapah S Roh TY Barski A Wang Z Dynamic regulation of nucleosome positioning in the human genome. Cell (2008) 132:887–98 10.1016/j.cell.2008.02.022 18329373 PMC10894452

[144] Harikrishnan KN Chow MZ Baker EK Pal S Bassal S Brasacchio D Brahma links the SWI/SNF chromatin-remodeling complex with MeCP2-dependent transcriptional silencing. Nat Genet (2005) 37:254–64 10.1038/ng1516 15696166

[145] Wysocka J Swigut T Xiao H Milne TA Kwon SY Landry J HD finger of NURF couples histone H3 lysine 4 trimethylation with chromatin remodelling. Nature (2006) 442:86–90 16728976 10.1038/nature04815

[146] Xia G Schneider-Stock R Diestel A Habold C Krueger S Roessner A *Helicobacter pylori* regulates p21(WAF1) by histone H4 acetylation. Biochem Biophys Res Commun (2008) 369:526–31 10.1016/j.bbrc.2008.02.073 18302936

[147] Kurdistani SK Histone modifications as markers of cancer prognosis: a cellular view. Br J Cancer (2007) 97:1–5 10.1038/sj.bjc.6603844 17592497 PMC2359665

[148] Fehri LF Rechner C Janssen S Mak TN Holland C Bartfeld S *Helicobacter pylori*-induced modification of the histone H3 phosphorylation status in gastric epithelial cells reflects its impact on cell cycle regulation. Epigenetics (2009) 4:577–86 10.4161/epi.4.8.10217 20081355

[149] Ding SZ Fischer W Kaparakis-Liaskos M Liechti G Merrell DS Grant PA *Helicobacter pylori*-induced histone modification, associated gene expression in gastric epithelial cells, and its implication in pathogenesis. PLoS One (2010) 5:e9875 10.1371/journal.pone.0009875 20368982 PMC2848570

[150] Angrisano T Lembo F Peluso S Keller S Chiariotti L Pero R *Helicobacter pylori* regulates iNOS promoter by histone modifications in human gastric epithelial cells. Med Microbiol Immunol (2012) 201:249–57 10.1007/s00430-011-0227-9 22215089

[151] Byun SW Chang YJ Chung IS Moss SF Kim SS *Helicobacter pylori* decreases p27 expression through the delta opioid receptor-mediated inhibition of histone acetylation within the p27 promoter. Cancer Lett (2012) 326:96–104 10.1016/j.canlet.2012.07.032 22867947 PMC3444678

[152] Ghildiyal M Zamore PD Small silencing RNAs: an expanding universe. Nat Rev Genet (2009) 10:94–108 10.1038/nrg2504 19148191 PMC2724769

[153] Xiao C Rajewsky K MicroRNA control in the immune system: basic principles. Cell (2009) 136:26–36 10.1016/j.cell.2008.12.027 19135886

[154] Link A Kupcinskas J Wex T Malfertheiner P Macro-role of microRNA in gastric cancer. Dig Dis (2012) 30:255–67 10.1159/000336919 22722550

[155] Yin Y Li J Chen S Zhou T Si J MicroRNAs as diagnostic biomarkers in gastric cancer. Int J Mol Sci (2012) 13:12544–55 10.3390/ijms131012544 23202912 PMC3497286

[156] Noto JM Peek RM The role of microRNAs in *Helicobacter pylori* pathogenesis and gastric carcinogenesis. Front Cell Infect Microbiol (2011) 1:21 10.3389/fcimb.2011.00021 22919587 PMC3417373

[157] Matsushima K Isomoto H Inoue N Nakayama T Hayashi T Nakayama M MicroRNA signatures in *Helicobacter pylori*-infected gastric mucosa. Int J Cancer (2011) 128:361–70 10.1002/ijc.25348 20333682

[158] Belair C Baud J Chabas S Sharma CM Vogel J Staedel C *Helicobacter pylori* interferes with an embryonic stem cell micro RNA cluster to block cell cycle progression. Silence (2011) 2:7 10.1186/1758-907X-2-7 22027184 PMC3212895

[159] Kato I Tominaga S Ito Y Kobayashi S Yoshii Y Matsuura A A prospective study of atrophic gastritis and stomach cancer risk. Jpn J Cancer Res (1992) 83:1137–42 10.1111/j.1349-7006.1992.tb02736.x 1483928 PMC5918704

[160] Vannella L Lahner E Annibale B Risk for gastric neoplasias in patients with chronic atrophic gastritis: a critical reappraisal. World J Gastroenterol (2012) 18:1279–85 10.3748/wjg.v18.i12.1279 22493541 PMC3319954

[161] Muller A Solnick JV Inflammation, immunity, and vaccine development for *Helicobacter pylori*. Helicobacter (2011) 16(Suppl 1):26–32 10.1111/j.1523-5378.2011.00877.x 21896082

[162] D’Elios MM Amedei A Benagiano M Azzurri A Del Prete G *Helicobacter pylori*, T cells and cytokines: the “dangerous liaisons.” FEMS Immunol Med Microbiol (2005) 44:113–9 10.1016/j.femsim.2004.10.013 15866204

[163] D’Elios MM Amedei A Del Prete G *Helicobacter pylori* antigen-specific T-cell responses at gastric level in chronic gastritis, peptic ulcer, gastric cancer and low-grade mucosa-associated lymphoid tissue (MALT) lymphoma. Microbes Infect (2003) 5:723–30 10.1016/S1286-4579(03)00114-X 12814773

[164] Garhart CA Redline RW Nedrud JG Czinn SJ Clearance of *Helicobacter pylori* infection and resolution of postimmunization gastritis in a kinetic study of prophylactically immunized mice. Infect Immun (2002) 70:3529–38 10.1128/IAI.70.7.3529-3538.2002 12065492 PMC128038

[165] Raghavan S Svennerholm AM Holmgren J Effects of oral vaccination and immunomodulation by cholera toxin on experimental *Helicobacter pylori* infection, reinfection, and gastritis. Infect Immun (2002) 70:4621–7 10.1128/IAI.70.8.4621-4627.2002 12117975 PMC128197

[166] Amieva MR El-Omar EM Host-bacterial interactions in *Helicobacter pylori* infection. Gastroenterology (2008) 134:306–23 10.1053/j.gastro.2007.11.009 18166359

[167] Sundquist M Quiding-Jarbrink M *Helicobacter pylori* and its effect on innate and adaptive immunity: new insights and vaccination strategies. Expert Rev Gastroenterol Hepatol (2010) 4:733–44 10.1586/egh.10.71 21108593

[168] Raghavan S Quiding-Jarbrink M Immune modulation by regulatory T cells in *Helicobacter pylori*-associated diseases. Endocr Metab Immune Disord Drug Targets (2012) 12:71–85 10.2174/187153012799278974 22214337

[169] Mandell L Moran AP Cocchiarella A Houghton J Taylor N Fox JG Intact Gram-negative *Helicobacter pylori, Helicobacter felis*, and *Helicobacter hepaticus* bacteria activate innate immunity via toll-like receptor 2 but not toll-like receptor 4. Infect Immun (2004) 72:6446–54 10.1128/IAI.72.11.6446-6454.2004 15501775 PMC523003

[170] Smith MF Jr Mitchell A Li G Ding S Fitzmaurice AM Ryan K Toll-like receptor (TLR) 2 and TLR5, but not TLR4, are required for *Helicobacter pylori*-induced NF-kappa B activation and chemokine expression by epithelial cells. J Biol Chem (2003) 278:32552–60 10.1074/jbc.M305536200 12807870

[171] Ding SZ Torok AM Smith MF Jr Goldberg JB Toll-like receptor 2-mediated gene expression in epithelial cells during *Helicobacter pylori* infection. Helicobacter (2005) 10:193–204 10.1111/j.1523-5378.2005.00311.x 15904477

[172] Kawahara T Kuwano Y Teshima-Kondo S Kawai T Nikawa T Kishi K Toll-like receptor 4 regulates gastric pit cell responses to *Helicobacter pylori* infection. J Med Invest (2001) 48:190–7 11694959

[173] Gewirtz AT Yu Y Krishna US Israel DA Lyons SL Peek RM Jr *Helicobacter pylori* flagellin evades toll-like receptor 5-mediated innate immunity. J Infect Dis (2004) 189:1914–20 10.1086/386289 15122529

[174] Amedei A Cappon A Codolo G Cabrelle A Polenghi A Benagiano M The neutrophil-activating protein of *Helicobacter pylori* promotes Th1 immune responses. J Clin Invest (2006) 116:1092–101 10.1172/JCI27177 16543949 PMC1401483

[175] Hold GL Rabkin CS Chow WH Smith MG Gammon MD Risch HA A functional polymorphism of toll-like receptor 4 gene increases risk of gastric carcinoma and its precursors. Gastroenterology (2007) 132:905–12 10.1053/j.gastro.2006.12.026 17324405

[176] Zeng HM Pan KF Zhang Y Zhang L Ma JL Zhou T Genetic variants of toll-like receptor 2 and 5, *Helicobacter pylori* infection, and risk of gastric cancer and its precursors in a Chinese population. Cancer Epidemiol Biomarkers Prev (2011) 20:2594–602 10.1158/1055-9965.EPI-11-0702 21994405

[177] Kupcinskas J Wex T Bornschein J Selgrad M Leja M Juozaityte E Lack of association between gene polymorphisms of angiotensin converting enzyme, Nod-like receptor 1, toll-like receptor 4, FAS/FASL and the presence of *Helicobacter pylori*-induced premalignant gastric lesions and gastric cancer in Caucasians. BMC Med Genet (2011) 12:112 10.1186/1471-2350-12-112 21864388 PMC3166912

[178] Pimentel-Nunes P Goncalves N Boal-Carvalho I Afonso L Lopes P Roncon-Albuquerque R Jr *Helicobacter pylori* induces increased expression of Toll-like receptors and decreased Toll-interacting protein in gastric mucosa that persists throughout gastric carcinogenesis. Helicobacter (2013) 18:22–32 10.1111/hel.12008 23061653

[179] Fukata M Abreu MT Role of toll-like receptors in gastrointestinal malignancies. Oncogene (2008) 27:234–43 10.1038/sj.onc.1210908 18176605 PMC2821878

[180] Kaparakis M Philpott DJ Ferrero RL Mammalian NLR proteins; discriminating foe from friend. Immunol Cell Biol (2007) 85:495–502 10.1038/sj.icb.7100105 17680011

[181] Shanks AM El-Omar EM *Helicobacter pylori* infection, host genetics and gastric cancer. J Dig Dis (2009) 10:157–64 10.1111/j.1751-2980.2009.00380.x 19659782

[182] Grubman A Kaparakis M Viala J Allison C Badea L Karrar A The innate immune molecule, NOD1, regulates direct killing of *Helicobacter pylori* by antimicrobial peptides. Cell Microbiol (2010) 12:626–39 10.1111/j.1462-5822.2009.01421.x 20039881

[183] Viala J Chaput C Boneca IG Cardona A Girardin SE Moran AP Nod1 responds to peptidoglycan delivered by the *Helicobacter pylori* cag pathogenicity island. Nat Immunol (2004) 5:1166–74 10.1038/ni1131 15489856

[184] Kim EJ Lee JR Chung WC Jung SH Sung HJ Lee YW Association between genetic polymorphisms of NOD 1 and *Helicobacter pylori*-induced gastric mucosal inflammation in healthy Korean population. Helicobacter (2013) 18:143–50 10.1111/hel.12020 23136938

[185] Zabaleta J Multifactorial etiology of gastric cancer. Methods Mol Biol (2012) 863:411–35 10.1007/978-1-61779-612-8_26 22359309 PMC3625139

[186] El-Omar EM Rabkin CS Gammon MD Vaughan TL Risch HA Schoenberg JB Increased risk of noncardia gastric cancer associated with proinflammatory cytokine gene polymorphisms. Gastroenterology (2003) 124:1193–201 10.1016/S0016-5085(03)00157-4 12730860

[187] El-Omar EM Carrington M Chow WH McColl KE Bream JH Young HA The role of interleukin-1 polymorphisms in the pathogenesis of gastric cancer. Nature (2001) 412:99 10.1038/35083629 11808612

[188] Zhang Y Liu C Peng H Zhang J Feng Q IL1 receptor antagonist gene IL1-RN variable number of tandem repeats polymorphism and cancer risk: a literature review and meta-analysis. PLoS One (2012) 7:e46017 10.1371/journal.pone.0046017 23049925 PMC3457944

[189] Santos JC Ladeira MS Pedrazzoli J Jr Ribeiro ML Relationship of IL-1 and TNF-alpha polymorphisms with *Helicobacter pylori* in gastric diseases in a Brazilian population. Braz J Med Biol Res (2012) 45:811–7 10.1590/S0100-879X2012007500099 22714811 PMC3854325

[190] Tahara T Shibata T Yamashita H Yoshioka D Okubo M Yonemura J Synergistic effect of IL-1beta and TNF-alpha polymorphisms on the *H. pylori*-related gastric pre-malignant condition. Hepatogastroenterology (2012) 59:2416–20 10.5754/hge10605 23169178

[191] Persson C Canedo P Machado JC El-Omar EM Forman D Polymorphisms in inflammatory response genes and their association with gastric cancer: a HuGE systematic review and meta-analyses. Am J Epidemiol (2011) 173:259–70 10.1093/aje/kwq370 21178102 PMC3105271

[192] Machado JC Figueiredo C Canedo P Pharoah P Carvalho R Nabais S A proinflammatory genetic profile increases the risk for chronic atrophic gastritis and gastric carcinoma. Gastroenterology (2003) 125:364–71 10.1016/S0016-5085(03)00899-0 12891537

[193] Partida-Rodriguez O Torres J Flores-Luna L Camorlinga M Nieves-Ramirez M Lazcano E Polymorphisms in TNF and HSP-70 show a significant association with gastric cancer and duodenal ulcer. Int J Cancer (2010) 126:1861–8 10.1002/ijc.24773 19626584

[194] Yea SS Yang YI Jang WH Lee YJ Bae HS Paik KH Association between TNF-alpha promoter polymorphism and *Helicobacter pylori* cagA subtype infection. J Clin Pathol (2001) 54:703–6 10.1136/jcp.54.9.703 11533078 PMC1731516

[195] Wu MS Wu CY Chen CJ Lin MT Shun CT Lin JT Interleukin-10 genotypes associate with the risk of gastric carcinoma in Taiwanese Chinese. Int J Cancer (2003) 104:617–23 10.1002/ijc.10987 12594817

[196] Kim J Cho YA Choi IJ Lee YS Kim SY Shin A Effects of interleukin-10 polymorphisms, *Helicobacter pylori* infection, and smoking on the risk of noncardia gastric cancer. PLoS One (2012) 7:e29643 10.1371/journal.pone.0029643 22235320 PMC3250465

[197] Tahara T Shibata T Arisawa T Nakamura M Yoshioka D Okubo M The BB genotype of heat-shock protein (HSP) 70-2 gene is associated with gastric pre-malignant condition in *H. pylori*-infected older patients. Anticancer Res (2009) 29:3453–8 19661373

[198] Hall TJ Role of hsp70 in cytokine production. Experientia (1994) 50:1048–53 10.1007/BF01923460 7988664

[199] Ohyauchi M Imatani A Yonechi M Asano N Miura A Iijima K The polymorphism interleukin 8 -251 A/T influences the susceptibility of *Helicobacter pylori* related gastric diseases in the Japanese population. Gut (2005) 54:330–5 10.1136/gut.2003.033050 15710978 PMC1774396

[200] Xue H Wang YC Lin B An J Chen L Chen J A meta-analysis of interleukin-10 -592 promoter polymorphism associated with gastric cancer risk. PLoS One (2012) 7:e39868 10.1371/journal.pone.0039868 22859944 PMC3409223

[201] McLean MH El-Omar EM Genetic aspects of inflammation. Curr Opin Pharmacol (2009) 9:370–4 10.1016/j.coph.2009.06.003 19570714

[202] Liu L Zhuang W Wang C Chen Z Wu XT Zhou Y Interleukin-8 -251 A/T gene polymorphism and gastric cancer susceptibility: a meta-analysis of epidemiological studies. Cytokine (2010) 50:328–34 10.1016/j.cyto.2010.03.008 20363644

[203] Amedei A Della Bella C Silvestri E Prisco D D’Elios MM T cells in gastric cancer: friends or foes. Clin Dev Immunol (2012) 2012:690571 10.1155/2012/690571 22693525 PMC3369415

[204] Lee HE Chae SW Lee YJ Kim MA Lee HS Lee BL Prognostic implications of type and density of tumour-infiltrating lymphocytes in gastric cancer. Br J Cancer (2008) 99:1704–11 10.1038/sj.bjc.6604738 18941457 PMC2584941

[205] Sakaguchi S Sakaguchi N Shimizu J Yamazaki S Sakihama T Itoh M Immunologic tolerance maintained by CD25+ CD4+ regulatory T cells: their common role in controlling autoimmunity, tumor immunity, and transplantation tolerance. Immunol Rev (2001) 182:18–32 10.1034/j.1600-065X.2001.1820102.x 11722621

[206] Mills KH Regulatory T cells: friend or foe in immunity to infection? Nat Rev Immunol (2004) 4:841–55 10.1038/nri1485 15516964

[207] Lundgren A Suri-Payer E Enarsson K Svennerholm AM Lundin BS *Helicobacter pylori*-specific CD4+ CD25high regulatory T cells suppress memory T-cell responses to *H. pylori* in infected individuals. Infect Immun (2003) 71:1755–62 10.1128/IAI.71.4.1755-1762.2003 12654789 PMC152046

[208] Cheng HH Tseng GY Yang HB Wang HJ Lin HJ Wang WC Increased numbers of Foxp3-positive regulatory T cells in gastritis, peptic ulcer and gastric adenocarcinoma. World J Gastroenterol (2012) 18:34–43 10.3748/wjg.v18.i1.34 22228968 PMC3251803

[209] Lundgren A Trollmo C Edebo A Svennerholm AM Lundin BS *Helicobacter pylori*-specific CD4+ T cells home to and accumulate in the human *Helicobacter pylori*-infected gastric mucosa. Infect Immun (2005) 73:5612–9 10.1128/IAI.73.9.5612-5619.2005 16113278 PMC1231054

[210] Lundgren A Stromberg E Sjoling A Lindholm C Enarsson K Edebo A Mucosal FOXP3-expressing CD4+ CD25high regulatory T cells in *Helicobacter pylori*-infected patients. Infect Immun (2005) 73:523–31 10.1128/IAI.73.1.523-531.2005 15618192 PMC538965

[211] Wu C Chen X Liu J Li MY Zhang ZQ Wang ZQ Moxifloxacin-containing triple therapy versus bismuth-containing quadruple therapy for second-line treatment of *Helicobacter pylori* infection: a meta-analysis. Helicobacter (2011) 16:131–8 10.1111/j.1523-5378.2011.00826.x 21435091

[212] Arnold IC Lee JY Amieva MR Roers A Flavell RA Sparwasser T Tolerance rather than immunity protects from *Helicobacter pylori*-induced gastric preneoplasia. Gastroenterology (2011) 140:199–209 10.1053/j.gastro.2010.06.047 20600031 PMC3380634

[213] Freire de Melo F Rocha AM Rocha GA Pedroso SH de Assis Batista S Fonseca de Castro LP A regulatory instead of an IL-17 T response predominates in *Helicobacter pylori*-associated gastritis in children. Microbes Infect (2012) 14:341–7 10.1016/j.micinf.2011.11.008 22155622

[214] Kido M Watanabe N Aoki N Iwamoto S Nishiura H Maruoka R Dual roles of CagA protein in *Helicobacter pylori*-induced chronic gastritis in mice. Biochem Biophys Res Commun (2011) 412:266–72 10.1016/j.bbrc.2011.07.081 21820415

[215] Ong SP Duggan A Eradication of *Helicobacter pylori* in clinical situations. Clin Exp Med (2004) 4:30–8 10.1007/s10238-004-0035-2 15598083

[216] Talley NJ Fock KM Moayyedi P Gastric cancer consensus conference recommends *Helicobacter pylori* screening and treatment in asymptomatic persons from high-risk populations to prevent gastric cancer. Am J Gastroenterol (2008) 103:510–4 10.1111/j.1572-0241.2008.01819.x 18341483

[217] Kosunen TU Pukkala E Sarna S Seppala K Aromaa A Knekt P Gastric cancers in Finnish patients after cure of *Helicobacter pylori* infection: a cohort study. Int J Cancer (2011) 128:433–9 10.1002/ijc.25337 20309944

[218] Selgrad M Bornschein J Rokkas T Malfertheiner P *Helicobacter pylori*: gastric cancer and extragastric intestinal malignancies. Helicobacter (2012) 17(Suppl 1):30–5 10.1111/j.1523-5378.2012.00980.x 22958153

[219] Graham DY Lu H Yamaoka Y A report card to grade *Helicobacter pylori* therapy. Helicobacter (2007) 12:275–8 10.1111/j.1523-5378.2007.00518.x 17669098

[220] Georgopoulos SD Papastergiou V Karatapanis S Current options for the treatment of *Helicobacter pylori*. Expert Opin Pharmacother (2013) 14:211–23 10.1517/14656566.2013.763926 23331077

[221] Selgrad M Bornschein J Malfertheiner P *Helicobacter pylori*: an infection with local complications and systemic effects. Dtsch Med Wochenschr (2011) 136:1790–5 10.1055/s-0031-1286104 21882136

[222] Ruggiero P *Helicobacter pylori* infection: what’s new. Curr Opin Infect Dis (2012) 25:337–44 10.1097/QCO.0b013e3283531f7c 22555448

[223] Iwanczak F Iwanczak B Treatment of *Helicobacter pylori* infection in the aspect of increasing antibiotic resistance. Adv Clin Exp Med (2012) 21:671–80 23356205

[224] Niv Y Hazazi R Waked A Lederfein T Achiel K *Helicobacter pylori* recurrence and infection rate in Israeli adults. Dig Dis Sci (2008) 53:1211–4 10.1007/s10620-007-0016-x 17939051

[225] Czinn SJ Blanchard T Vaccinating against *Helicobacter pylori* infection. Nat Rev Gastroenterol Hepatol (2011) 8:133–40 10.1038/nrgastro.2011.1 21304478

[226] Aebischer T Bumann D Epple HJ Metzger W Schneider T Cherepnev G Correlation of T cell response and bacterial clearance in human volunteers challenged with *Helicobacter pylori* revealed by randomised controlled vaccination with Ty21a-based *Salmonella* vaccines. Gut (2008) 57:1065–72 10.1136/gut.2007.145839 18417532 PMC2564837

[227] Malfertheiner P Schultze V Rosenkranz B Kaufmann SH Ulrichs T Novicki D Safety and immunogenicity of an intramuscular *Helicobacter pylori* vaccine in noninfected volunteers: a phase I study. Gastroenterology (2008) 135:787–95 10.1053/j.gastro.2008.05.054 18619971

[228] Sutton P Chionh YT Why can’t we make an effective vaccine against *Helicobacter pylori*? Expert Rev Vaccines (2013) 12:433–41 10.1586/erv.13.20 23560923

[229] Atherton JC Blaser MJ Coadaptation of *Helicobacter pylori* and humans: ancient history, modern implications. J Clin Invest (2009) 119:2475–87 10.1172/JCI38605 19729845 PMC2735910

[230] Arnold IC Hitzler I Muller A The immunomodulatory properties of *Helicobacter pylori* confer protection against allergic and chronic inflammatory disorders. Front Cell Infect Microbiol (2012) 2:10 10.3389/fcimb.2012.00010 22919602 PMC3417532

[231] Ciortescu I Stan M *Helicobacter pylori* – friend or foe? Rev Med Chir Soc Med Nat Iasi (2010) 114:619–24 21243784

[232] Correa P *Helicobacter pylori* and gastric cancer: state of the art. Cancer Epidemiol Biomarkers Prev (1996) 5:477–81 8781746

[233] Hunt RH Will eradication of *Helicobacter pylori* infection influence the risk of gastric cancer? Am J Med (2004) 117(Suppl 5A):86S–91 15478858 10.1016/j.amjmed.2004.07.030

[234] Fall K Ye W Nyren O Antibiotic treatment and risk of gastric cancer. Gut (2006) 55:793–6 10.1136/gut.2006.091850 16551654 PMC1856225

[235] Fuccio L Eusebi LH Zagari RM Bazzoli F *Helicobacter pylori* eradication treatment reduces but does not abolish the risk of gastric cancer. Am J Gastroenterol (2009) 104(3100): author reply 3101-2, 10.1038/ajg.2009.516 19956126

[236] de Vries AC Kuipers EJ Rauws EA *Helicobacter pylori* eradication and gastric cancer: when is the horse out of the barn? Am J Gastroenterol (2009) 104:1342–5 10.1038/ajg.2008.15 19491846

[237] Wong BC Lam SK Wong WM Chen JS Zheng TT Feng RE *Helicobacter pylori* eradication to prevent gastric cancer in a high-risk region of China: a randomized controlled trial. JAMA (2004) 291:187–94 10.1001/jama.291.2.187 14722144

[238] Chang MH Decreasing incidence of hepatocellular carcinoma among children following universal hepatitis B immunization. Liver Int (2003) 23:309–14 10.1034/j.1478-3231.2003.00865.x 14708890

[239] Harper DM Vierthaler SL Next generation cancer protection: the bivalent HPV vaccine for females. ISRN Obstet Gynecol (2011) 2011:457204 10.5402/2011/457204 22111017 PMC3216348

